# Characterization of Aspergillus fumigatus secretome during sublethal infection of Galleria mellonella larvae

**DOI:** 10.1099/jmm.0.001844

**Published:** 2024-06-05

**Authors:** Aaron Curtis, Pavel Dobes, Jacek Marciniak, Jana Hurychova, Pavel Hyrsl, Kevin Kavanagh

**Affiliations:** 1 Department of Biology, Maynooth University, Maynooth, Co. Kildare, Ireland; 2 Department of Experimental Biology, Faculty of Science, Masaryk University, Kamenice 5, 625 00 Brno, Czech Republic

**Keywords:** *Aspergillus*, fungal–host interactions, *Galleria mellonella*, gliotoxin, proteomics

## Abstract

**Introduction.** The fungal pathogen *Aspergillus fumigatus* can induce prolonged colonization of the lungs of susceptible patients, resulting in conditions such as allergic bronchopulmonary aspergillosis and chronic pulmonary aspergillosis.

**Hypothesis.** Analysis of the *A. fumigatus* secretome released during sub-lethal infection of *G. mellonella* larvae may give an insight into products released during prolonged human colonisation.

**Methodology.***Galleria mellonella* larvae were infected with *A. fumigatus,* and the metabolism of host carbohydrate and proteins and production of fungal virulence factors were analysed. Label-free qualitative proteomic analysis was performed to identify fungal proteins in larvae at 96 hours post-infection and also to identify changes in the *Galleria* proteome as a result of infection.

**Results.** Infected larvae demonstrated increasing concentrations of gliotoxin and siderophore and displayed reduced amounts of haemolymph carbohydrate and protein. Fungal proteins (399) were detected by qualitative proteomic analysis in cell-free haemolymph at 96 hours and could be categorized into seven groups, including virulence (*n* = 25), stress response (*n* = 34), DNA repair and replication (*n* = 39), translation (*n* = 22), metabolism (*n* = 42), released intracellular (*n* = 28) and cellular development and cell cycle (*n* = 53). Analysis of the Gallerial proteome at 96 hours post-infection revealed changes in the abundance of proteins associated with immune function, metabolism, cellular structure, insect development, transcription/translation and detoxification.

**Conclusion.** Characterizing the impact of the fungal secretome on the host may provide an insight into how *A. fumigatus* damages tissue and suppresses the immune response during long-term pulmonary colonization.

## Introduction

*Aspergillus fumigatus* is a ubiquitous environmental fungus and a significant pathogen capable of producing a variety of pulmonary infections in susceptible patients [1]. The most serious form of infection is invasive aspergillosis, and this can induce a mortality rate of 50 % in neutropenic patients and 90 % in stem cell therapy recipients [2]. Prolonged fungal colonization is a common characteristic of allergic bronchopulmonary aspergillosis (ABPA), which typically affects those with hyperactive immune responses, such as asthma or cystic fibrosis [3]. ABPA is characterized by repeated exacerbations arising from *Aspergillus* sensitization resulting in severe immune response and inflammation leading to haemoptysis or, in severe cases, lung collapse [4]. The development of ABPA depends upon fungal persistence and non-lethal colonization despite intense inflammatory cell infiltration driven by a number of fungal virulence factors including secreted proteases capable of detaching cells from the basement membrane leading to altered epithelial integrity [5]. In the case of pre-existing lung damage or modest immunosuppression, chronic pulmonary aspergillosis (CPA) can occur. CPA is characterized by slow progressive destruction of lung parenchyma and recurrence upon discontinuation of antifungal therapy. The condition often results in cavity formation or expansion of pre-existing cavities from previous insults [6]. Damage caused to the lung tissue, commonly following tuberculosis infection, facilitates saprophytic colonization [7].

*A. fumigatus* produces a range of enzymes, toxins and small molecules that facilitate the growth and survival of the fungus in the environment, and these may facilitate persistence in the host. The thermotolerance of *A. fumigatus* may have evolved to allow survival in its environmental niche, and this is facilitated by the thick conidia walls coupled with transcriptional regulation of heat shock response proteins in response to the loss of cell wall integrity [8]. *A. fumigatus* produces low-molecular-mass ferric-iron-specific chelators known as siderophores, which are upregulated during iron starvation and are integral to fungal virulence [9]. Siderophores such as fusarine C, and its acetylated form triacetylfusarine C, capture extracellular iron, while ferricrocin may be intracellular and involved in iron distribution and storage [10]. The conidial siderophore hydroxyferricrocin plays a crucial role in iron storage, germination and oxidative stress resistance [11]. Gliotoxin, an immunosuppressive epipolythiodioxopiperzine toxin, can induce apoptosis in neutrophils, which are an important part of the immune response to fungal infection [12]. Gliotoxin and fumagillin may damage lung epithelial cells by producing reactive oxygen species [13].

*Galleria mellonella* larvae are widely used for assessing the virulence of bacterial [14] and fungal [15] pathogens and for assessing the *in vivo* efficacy and toxicity of antimicrobial agents.[16] The use of *G. mellonella* larvae provides results comparable to those obtained using mammals [17,18] due to the many structural and functional similarities between the insect immune response and the innate immune response of mammals [19]. Such similarities include the presence of pattern recognition receptors, which can induce signalling cascades initiating cellular and humoral immune responses such as phagocytosis, nodulation, agglutination, encapsulation, coagulation and the production of antimicrobial peptides and enzymes [20]. Larvae can be maintained at 37 °C, enabling analysis of temperature-dependent virulence factors [21]. *G. mellonella* larvae are susceptible to infection with a variety of fungal pathogens, including *Candida albicans* [22], *Madurella mycetomatis* [23] and *A. fumigatus* [24], and symptoms demonstrate strong similarities to those evident during mammalian infection.

While *G. mellonella* larvae have many advantages as a convenient, easy-to-use *in vivo* model, previous work has often used lethal doses of pathogens [25,26]. In the present work, the secretome produced by *A. fumigatus* during sublethal colonization of * G. mellonella* larvae was monitored as this might provide an insight into the processes that occur during long-term colonization of human tissue by *A. fumigatus*.

## Methods

### *A. fumigatus* culture conditions

*A. fumigatus* ATCC 26933 was cultured for 72 hours at 37 °C on Malt extract agar (Oxoid) plates following point inoculation. Conidia were harvested by washing plates with sterile PBS supplemented with 0.1 % (v/v) Tween 20 (Sigma Aldrich, USA) and enumerated using a haemocytometer.

### Virulence assessment of *A. fumigatus in vivo*

Sixth instar larvae of *G. mellonella* (Livefoods Direct Ltd, Sheffield, England) were stored at 15 °C prior to use. Larvae (*n* = 20) weighing 0.2–0.3 g without signs of melanization were inoculated with 20 µl PBS containing 1×10^5^, 1×10^6^ or 1×10^7^ *A. fumigatus* conidia via intra-haemocoel injection using a 26G 1 ml syringe (Terumo). Larvae were placed in 9 cm petri dishes and incubated at 37 °C. Larval viability was monitored over 96 hours. In all subsequent experiments, 1×10^5^ conidia/larva was used as the inoculation dose.

### Assessment of larval movement

The movement of larvae infected with 1×10^5^ *A. fumigatus* conidia at 24, 48, 72 and 96 hours post-infection was assessed via the FIMTrack method [27,28] using frustrated total internal reflection of infrared light in acrylic glass. Images were captured via Basler acA2040-90uc camera in a dark room and with a frequency of one frame per second for 600 s. The scale factor was 80 pixels/cm. Images were processed by FIMTrack v2 Windows (×86) software (downloaded from http://fim.uni-muenster.de/). Data gathered from the software were processed and visualized in Prism 8.0.1 (USA GraphPad).

### Quantification of total protein and carbohydrate in larval haemolymph

Haemolymph was extracted from larvae infected with 1×10^5^ *A. fumigatus* conidia at 24-hour intervals over 96 hours and diluted 50× in Milli-Q water. The total protein concentration was measured according to the Lowry method using a commercial kit (DC Protein Assay, Bio-Rad, Hercules, CA, USA). Bovine serum albumin (Sigma-Aldrich, St. Louis, MO, USA) was used as a standard, and the absorbance was measured with Multiscan GO (ThermoFisher Scientific, Waltham, MA, USA) spectrophotometer at 700 nm. The concentration of total carbohydrates was determined by the anthrone method [29]. Specifically, 50 µl of 40× diluted haemolymph was used per reaction, and absorbance was measured at 620 nm with a spectrophotometer Sense (Hidex, Turku, Finland). The concentration of total carbohydrates was calculated according to a calibration curve prepared with glucose (Sigma-Aldrich, St. Louis, MO, USA) as a standard.

### *In vivo* gliotoxin extraction and quantification

*G. mellonella* larvae were infected with 1×10^5^ *A. fumigatus* conidia. Larvae (*n* = 25) were flash frozen in liquid nitrogen at 24, 48, 72 and 96 hours post-infection and ground using a mortar and pestle. The material was transferred with 5 ml of 6 M hydrochloric acid (HCl) to a centrifuge tube, and the mortar was washed twice with 5 ml volumes of HCl. Chloroform (25 ml) was added to the tube, which was mixed constantly for 30 min. Chloroform fraction was extracted and mixed with another 25 ml of chloroform; the process was repeated, and the chloroform fractions were pooled. The chloroform fraction was stored at −20 °C overnight, and the lipid fraction was removed. The samples were dried in a Büchi rotor evaporator (Brinkmann Instruments). Samples were dissolved in 500 µl methanol and stored at −20 °C for further use. Gliotoxin was detected by reverse-phase HPLC (Shimadzu) and quantified through the generation of a standard curve using gliotoxin standards (100, 50, 25, 12.5 and 6.25 µg ml^−1^) dissolved in methanol. The mobile phase consisted of 34.9 % (v/v) acetonitrile (Fisher Scientific), 0.1 % (v/v) trifluoroacetic acid (Sigma Aldrich) and 65 % (v/v) HPLC-grade water (ddH_2_O). Sample (20 µl) was loaded onto an Agilent ZORBAX SB-Aq 5 µm polar LC column.

### Total secreted siderophore quantification

Siderophore activity in haemolymph was determined using SideroTec HiSens assay (Accuplex, www.accuplexdiagnostics.comwww.accuplexdiagnostics.com). Briefly, 100 µl of haemolymph from infected larvae (*n* = 3) diluted 1/10 in PBS was added to a 96-well microplate followed by the addition of 100 µl of the ready-to-use detector. After 10 min incubation at 37 °C, fluorescence was measured on a fluorescence reader (Bio-Tek Synergy HT) using the emission/excitation filter 360/460 nm. Siderophore concentration was quantified using deferoxamine as a reference standard.

### Protein extraction

*G. mellonella* larvae (*n* = 3) infected with 1×10^5^ *A. fumigatus* conidia and a PBS control were bled via the third left thoracic leg at 96 hours post-infection yielding 40 µl haemolymph per larva. The pooled haemolymph was centrifuged at 10 000 ***g***, and the cell-free haemolymph was diluted 1/5 in sterile PBS. Protein concentration was determined using the Qubit quantification system (Invitrogen, Waltham, MA, USA). An aliquot containing 55 µg of protein was purified and digested using filter-aided sample preparation [30]. Briefly, samples were mixed with 200 µl 8 M urea in the filter unit and spun at 14 000 ***g*** for 30 min. An additional 200 µl was added and spun again, and the flowthrough was discarded. Iodoacetamide (100 µl, 0.05 M) was added, and samples were mixed at 600 r.p.m. in a thermomixer for 1 min and incubated for 20 min at room temperature without mixing. Urea (100 µl) was added, and samples were centrifuged at 14 000 ***g*** for 15 min; this step was repeated. Ammonium bicarbonate (100 µl, 0.05 M) was added, and samples were centrifuged at 14 000 ***g*** for 15 min; this step was repeated. A digestion buffer containing 0.4 µg ml^−1^ trypsin, 0.05 % protease max and 0.05 M ammonium bicarbonate was added to give a final trypsin to protein ratio of 1 : 40. Samples were incubated for 18 hours in a humidified chamber at 37 °C. Samples were transferred to fresh collection tubes; 40 µl ammonium bicarbonate was added, and the samples were centrifuged for 10 min at 14,000 ***g*** and acidified with acidification buffer (1 : 10 ratio; 78 % LC-MS-grade water, 20 % acetonitrile and 2 % trifluoracetic acid).

### Proteomic analysis

Of the digested protein, 600 ng was loaded onto a Q Exactive (ThermoFisher Scientific) high-resolution mass spectrometer, which was connected to a Dionex Ultimate 3000 (RSlCnano) chromatography system. An increasing acetonitrile gradient was used to separate the peptides in the samples. This gradient was created on a 50-cm EASY-Spray PepMap C18 column with a 75 mm diameter using a 133-min reverse-phase gradient at a flow rate of 300 nl min^−1^. The data were obtained, while the mass spectrometer was functioning in an automatic-dependent switching mode.

Qualitative analysis of the fungal and larval protein content of the cell-free haemolymph was investigated using Proteome Discoverer 2.5 and Sequest HT (SEQUEST HT algorithm; Thermo Scientific). Identified proteins were searched against the UniProtKB database *A. fumigatus,* 9647 entries, (UP000002530) and*G. mellonella,* 18 342 entries, (UP000504614). Search parameters applied for protein identification were as follows: (i) enzyme name – trypsin, (ii) an allowance of up to two missed cleavages, (iii) peptide mass tolerance set to 10 ppm, (iv) MS/MS mass tolerance set to 0.02 Da, (v) carbamidomethylation set as a fixed modification and (vi) methionine oxidation set as a variable modification. Peptide probability was set to high confidence (with an FDR ≤0.01 % as determined by Percolator validation in Proteome Discoverer). Peptides identified by two or more unique peptides were retained for analysis.

### Data analysis

Data gathered from the analyses were processed and visualized in Prism 8.0.1 (USA GraphPad). Survival curves of *Aspergillus*-infected larvae were compared to the survival of the PBS-injected group using the Mantel–Cox test. All other data were assessed for normality and homogeneity of variance, followed by one-way ANOVA with post hoc Dunnett’s test comparing data from all treated groups to the control group. The changes in proteins and carbohydrates between PBS-injected and *Aspergillus*-injected groups were compared by unpaired t-tests at each time of sample collection. Results with *P*-values less than 0.05 were considered statistically significant.

## Results

### Virulence of *A. fumigatus* in *G. mellonella* larvae

**Fig. 1.**
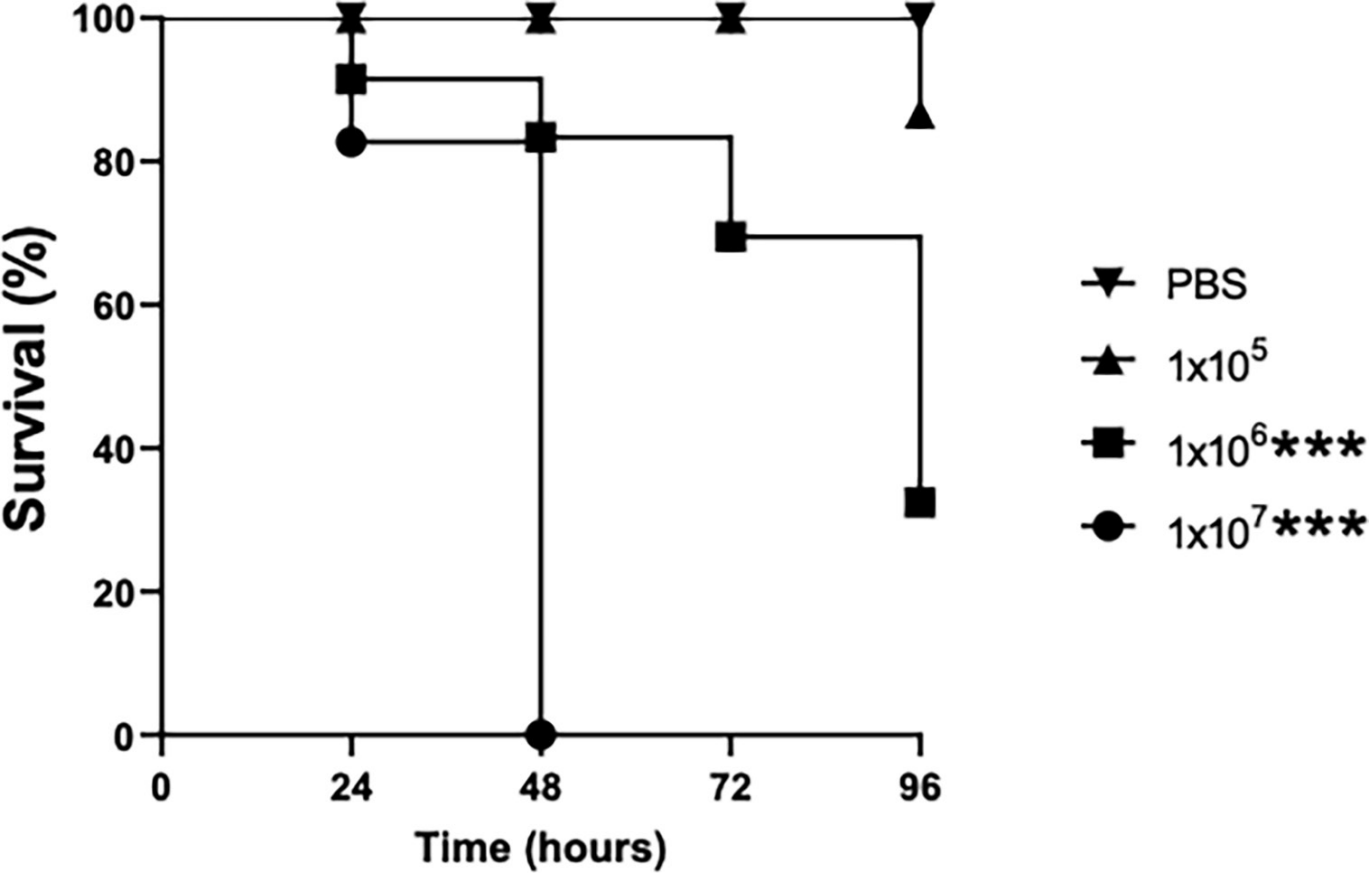
Dose-dependent survival of *G. mellonella* larvae infected with *A. fumigatus,* demonstrating a significant reduction in survival at 96 hours at concentrations of 1×10^6^ and 1×10^7^ (***P < 0.001) determined by log-rank (Mantel–Cox) test.

*G. mellonella* larvae were infected with *A. fumigatus* conidia at an initial dose of 1×10^5^, 1×10^6^ or 1×10^7^ per larva and incubated at 37 °C for 96 hours. Larvae infected with 1×10^7^ *A. fumigatus* conidia showed 0 % survival at 48 hours, while those infected with 1×10^6^ conidia/larva showed 85 % (17/20) survival at 48 hours and 35 % (7/20) survival at 96 hours. Larvae infected with 1×10^5^ conidia/larva showed 100 % (20/20) survival at 48 hours and 90 % (18/20) survival at 96 hours (Fig. 1).

**Fig. 2.**
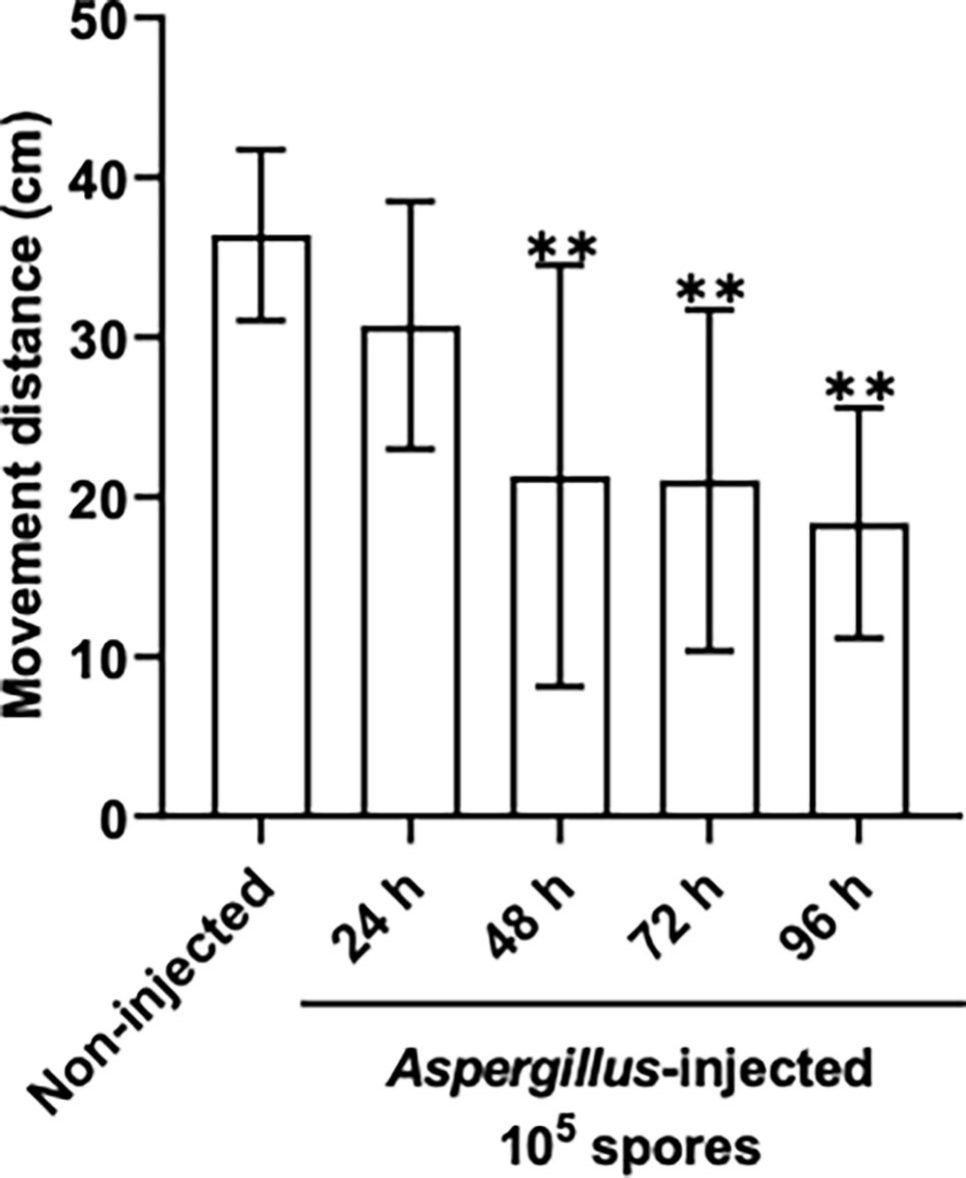
FIMTrack analysis of larval movement over time following infection with 1×10^5^ conidia, demonstrating a significant reduction in larval movement at all timepoints post 48 hours (*P* < 0.05) determined by Dunnett’s multiple comparisons test.

FIMTrack analysis was performed to monitor the activity of larvae infected with a dose of 1×10^5^ conidia/larva. The results showed that after infection, the larvae continued to move; however, the rate of movement was significantly reduced at 48 (*P* = 0.0046), 72 (*P* = 0.0038) and 96 hours (*P* = 0.0018) when compared to control larvae (Fig. 2). For all subsequent experiments, only larvae that had been infected with 1×10^5^ conidia/larva and showing signs of viability at 96 hours were used.

### Metabolite concentrations in larvae post *A. fumigatus* infection

**Fig. 3.**
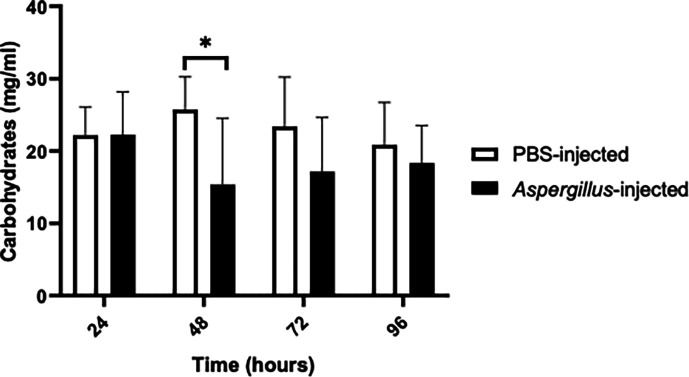
Assessment of haemolymph carbohydrate content post-infection with 1×10^5^ *A. fumigatus* conidia. Significant reduction in host carbohydrate content 48 hours post-infection (*P* = 0.0003) determined by unpaired t-test (*n* = 7–15 individual larvae per group). The experiment was conducted in three independent replicates.

**Fig. 4.**
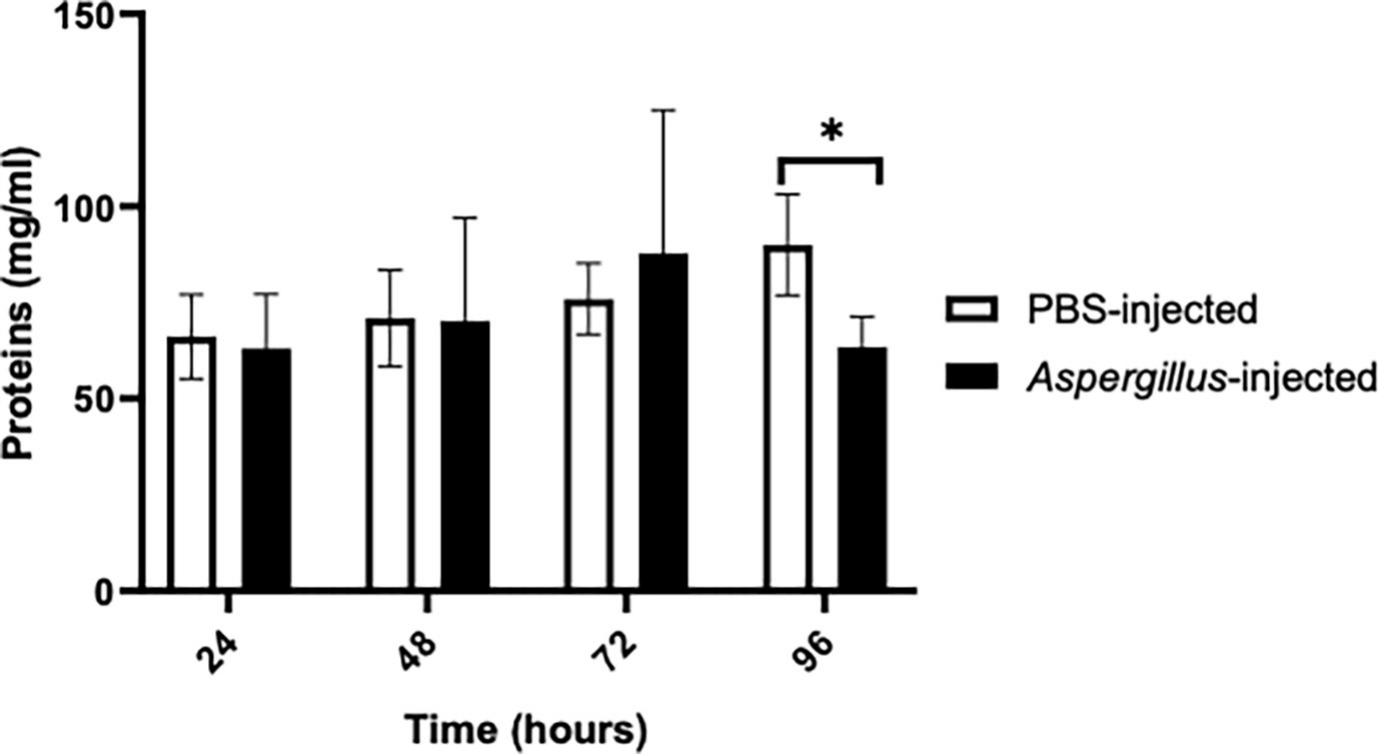
Assessment of haemolymph protein content post-infection with 1×10^5^ *A. fumigatus* conidia. Significant reduction in host protein content 96 hours post-infection (*P* = 0.0001) determined by unpaired t-test (*n* = 7–15 individual larvae per group). The experiment was conducted in three independent replicates.

The carbohydrate concentration of haemolymph collected from larvae infected with 1×10^5^ conidia/larva was lower than that of control larvae at 48 (*P* = 0.000373), 72 and 96 hours (Fig. 3). However, the protein content remained relatively constant until showing a statistically significant decrease at 96 hours (*P* = 0.000124) (Fig. 4).

### Gliotoxin quantification in *G. mellonella* larvae post *A. fumigatus* infection

**Fig. 5.**
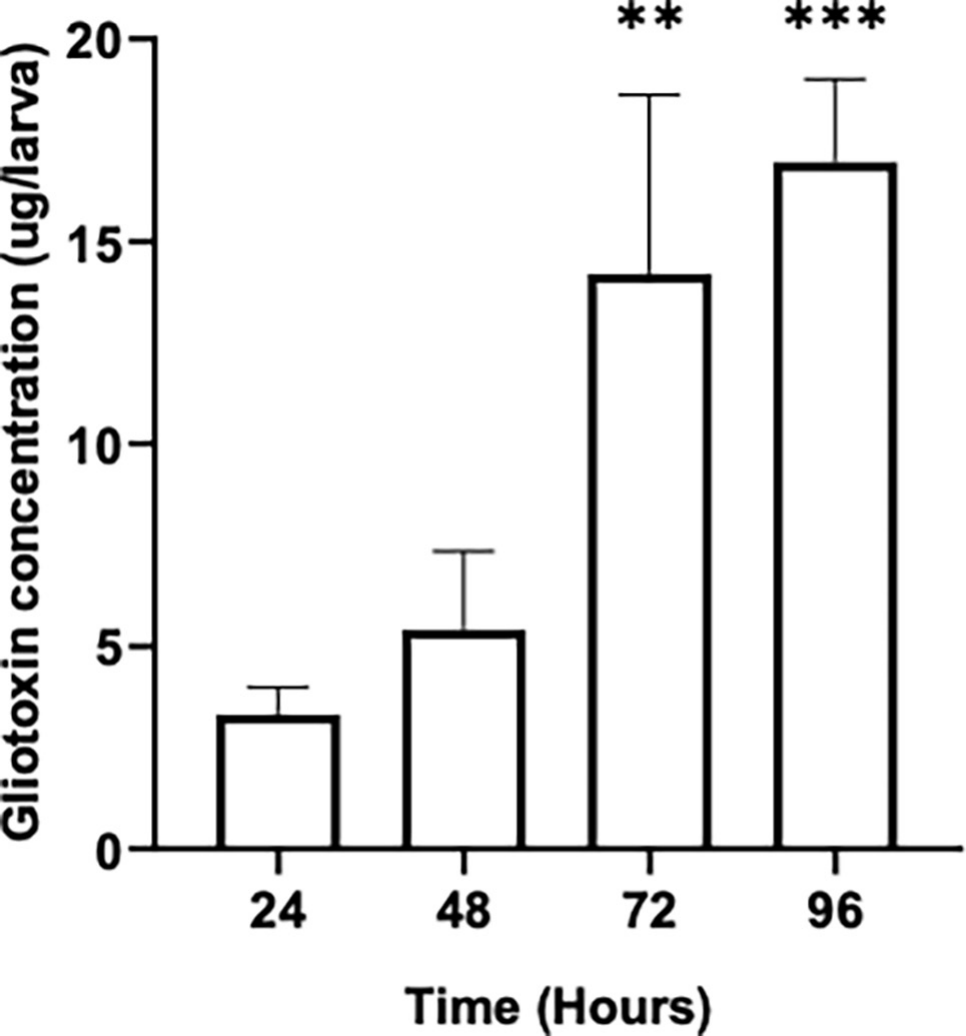
Quantification of gliotoxin in *Galleria* infected with 1×10^5^ *A. fumigatus* conidia, demonstrating significant increase production at 72 (**P = 0.02) and 96 (***P = 0.0006) hours post-infection when compared to the initial detection of 24 hours post-infection determined by Dunnett’s multiple comparisons test.

The gliotoxin concentration in larvae infected with 1×10^5^ conidia/larva was determined and indicated a steady increase over the course of the experiment with a concentration of 5.41 ± 1.57 µg/larva being recorded at 48 hours and 16.96±1.66 µg/larva 96 hours (*P* = 0.0003) (Fig. 5).

### Siderophore quantification in *G. mellonella* larvae post *A. fumigatus* infection

**Fig. 6.**
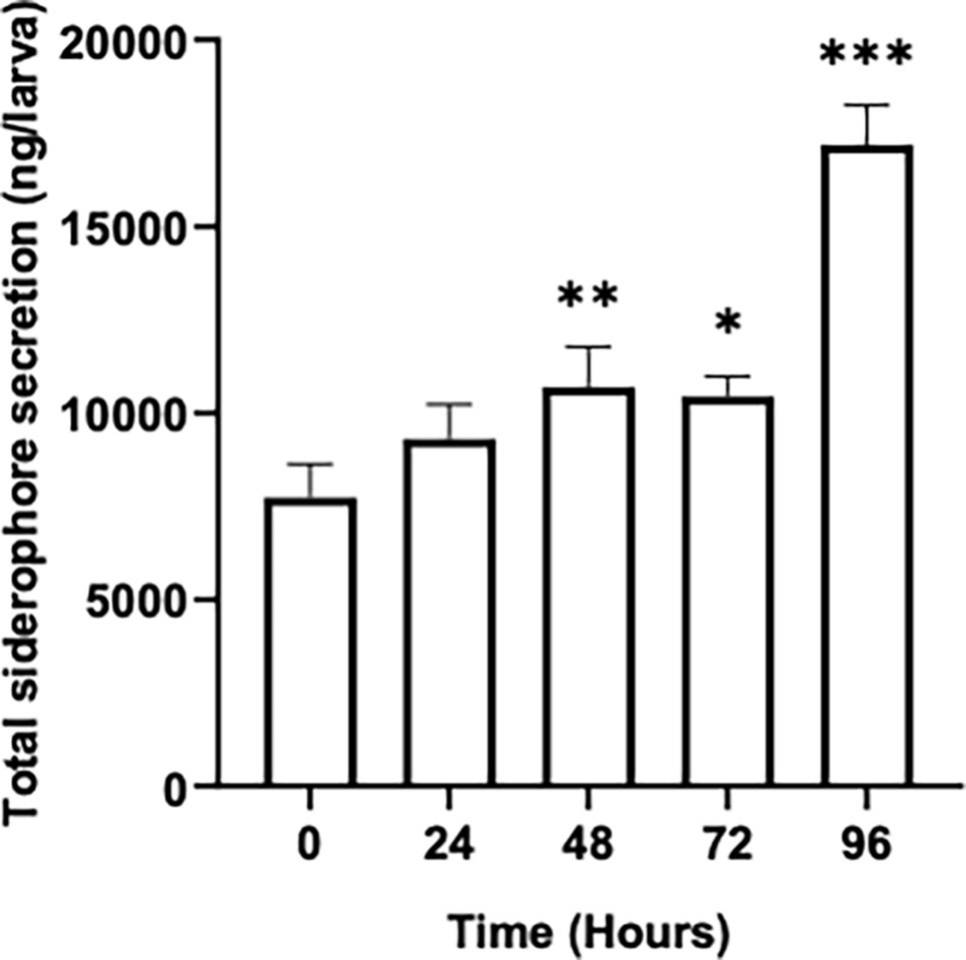
Quantification of total siderophore concentration detected in *Galleria* haemolymph infected with 1×10^5^ *A. fumigatus* conidia, demonstrating significantly increased production at 48 hours (*P* = 0.009) and all subsequent timepoints post-infection as determined by Dunnett’s multiple comparisons test.

The fungal siderophore concentration was also assessed and revealed a concentration of 5.54 ± 0.33 µg/larva at 48 hours and 12.59 ± 0.70 µg/larva at 96 hours (*P* < 0.0001) (Fig. 6).

### Proteomics analysis of *A. fumigatus-*infected larva

**Fig. 7.**
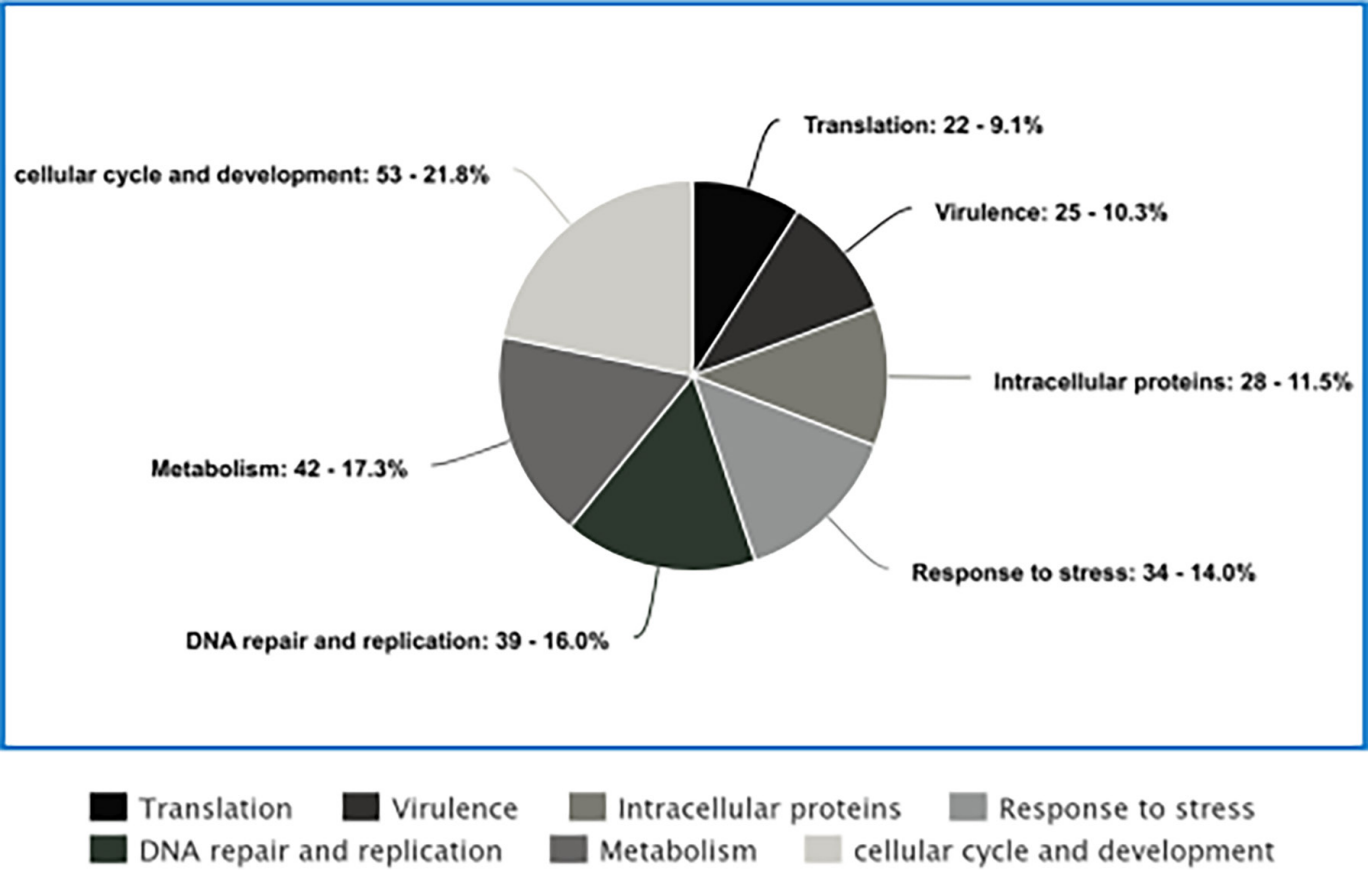
Pie chart summarizing high-confidence qualitative *A. fumigatus* protein detections categorized into seven subcategories: virulence (*n* = 25), stress response (*n* = 34), DNA repair and replication (*n* = 39), translation (*n* = 22), metabolism (*n* = 42), released intracellular (*n* = 28) and cellular cycle and development (*n* = 53) proteins.

Qualitative proteomic analysis of cell-free haemolymph from control larvae revealed 22 *Aspergillus* proteins, which were removed from subsequent analysis (Table S1, available in the online Supplementary Material), whereas larvae infected with 1×10^5^ conidia/larva for 96 hours revealed 339 *A*. *fumigatus* proteins (>2 unique peptide hits). Of these, 243 were well characterized and could be divided into 7 categories with assigned functions (Fig. 7). Virulence-associated proteins (*n* = 25) (Table S2.1) included non-ribosomal peptide synthase 13, O-methyltransferase af390-400 and dual-functional monooxygenase/methyltransferase psoF. Stress response proteins (*n* = 34) (Table S2.2) included proteins associated with environmental and drug-mediated stress, including glutathione S-transferase, HSP 70, PAB1-binding protein and ABC multidrug transporter A-2, atrI and H. Proteins associated with DNA repair/replication (*n* = 39) (Table S2.3) included fungal-specific transcription factor, kinetochore protein fta7, DNA polymerase and RAD52 DNA repair protein RDAC. Proteins associated with translation (*n* = 22) (Table S2.4) included elongation factor G, RNA-binding protein and RNA-dependent RNA polymerase. Proteins associated with metabolism (*n* = 42) (Table S2.5) included pyruvate carboxylase, trehalase, UTP-glucose-1-phosphate uridylyltransferase and tryptophan synthase. Released intracellular proteins (*n* = 28) (Table S2.6) include mitochondrial carrier protein, nuclear pore complex subunit NUP 192 and vacuolar ABC heavy metal transporter. Proteins associated with the cell cycle/cell development (*n* = 53) (Table S2.7) included cell cycle checkpoint protein Rad 17, meiosis protein ME12 and chitin synthase. Many of the proteins identified at this timepoint were associated with fungal secondary metabolism: non-ribosomal peptide synthetase 13, O-methyltransferase af390-400, pentafunctional AROM polypeptide, toxin biosynthesis protein (Tri7), putative and cytochrome P450 monooxygenase helB2.

### Proteomics analysis of *G. mellonella* response to infection

**Fig. 8.**
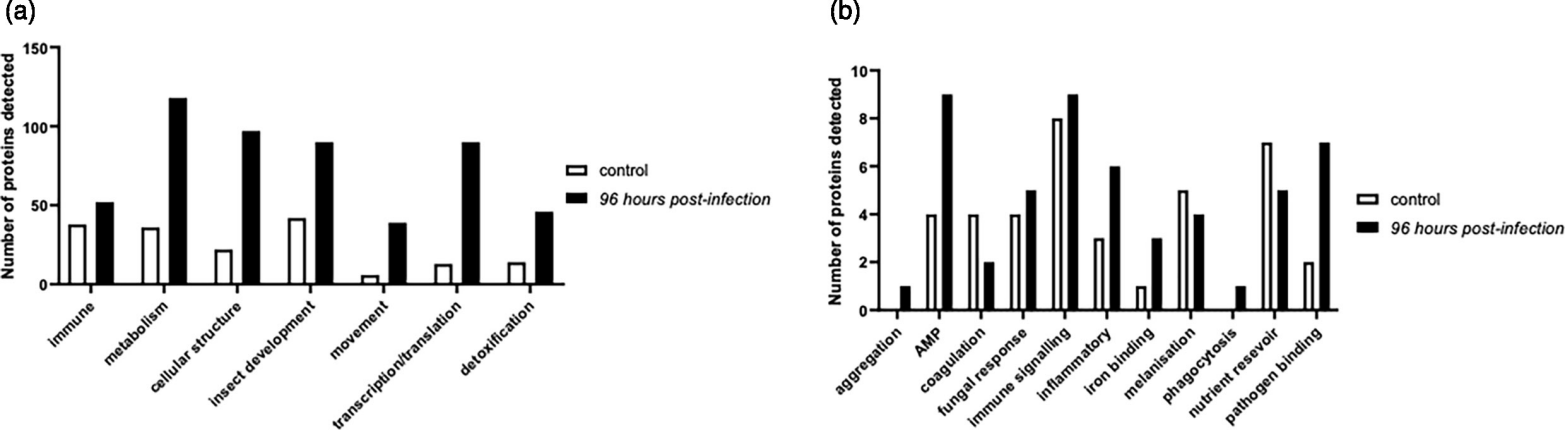
Bar graph summarizing (a) high-confidence qualitative *G. mellonella* protein detections categorized into seven subcategories in control larvae and in larvae at 96 hours post-infection. (b) Immune proteins further subdivided into 11 subcategories, and the greatest differences in protein abundance between the control and infected larvae were AMP (antimicrobial peptides), inflammation, nutrient reservoir and pathogen binding.

Qualitative proteomic analysis of cell-free haemolymph from control larvae revealed 215 *G*. *mellonella* proteins (>2 unique peptide hits), and of these, 171 were well characterized (Table S3.1a). In contrast, larvae infected with 1×10^5^ conidia/larva for 96 hours revealed 671 proteins, of which 532 were well characterized (Table S3.2a). Identified proteins could be divided into seven categories with assigned functions: immune function (38 in control, 52 in infected), metabolism (36 in control, 118 in infected), cellular structure (22 in control, 97 in infected), insect development (42 in control, 90 in infected), movement (6 in control, 39 in infected), transcription/translation (13 in control, 90 in infected) and detoxification (14 in control, 46 in infected) (Fig. 8a). Due to the importance of these proteins in the context of infection, the immune function proteins were further subdivided into 11 subcategories, and the greatest differences in protein abundance between the control and infected larvae were AMP(antimicrobial peptides) (4 control, 9 infected larva), inflammation (3 control, 6 infected), nutrient reservoir (7 control, 5 infected) and pathogen binding (2 control, 7 infected) (Fig. 8b, Tables S3.1b and S3.2b).

## Discussion

Prolonged colonization of pulmonary tissue is a feature of ABPA and CPA, and results in exaggerated immune responses and tissue damage [31,32]. In the work presented here, *G. mellonella* larvae were infected with a sublethal dose of *A. fumigatus* conidia, and the development of the fungus within the host was monitored as it might give an insight into the fungal–host interactions during prolonged colonization of human tissue. Infection of larvae with a dose of 1×10^5^ conidia/larva resulted in only 10 % mortality at 96 hours (Fig. 1). The remaining live larvae continued to move, although at a slower rate, when compared to the control cohort (Fig. 2). Fungal colonization of larvae resulted in the production of gliotoxin and siderophore, and the metabolism of host carbohydrates and proteins in the haemolymph. Qualitative proteomic analysis revealed a wide range of fungal proteins in the cell-free haemolymph at 96 hours, and these may have been released from the cell wall/conidia surface (e.g. beta-N-acetylglucosaminidase putative and chitin synthase) produced as a result of hyphal lysis (e.g. nuclear pore complex subunit Nup192 and sorting nexin-4) or secreted (e.g. pectin methylesterase), in addition to those released as a result of innate immune killing by the host. Understanding the impact of these and other proteins on the host may provide new insight into fungal–host interaction.

Hyphal development by *A. fumigatus* has been associated with the induction of inflammation, increased CD4/CD8 ratio and Th2 cell differentiation, promoting a proinflammatory environment [33]. ABPA is characterized by an exaggerated Th2-mediated response, triggering the release of inflammatory cytokines and growth factors leading to airway hyperresponsiveness, goblet cell hyperplasia and subepithelial fibrosis [34]. The detection of class V myosin (Myo4), putative (Table S2.7) at 96 hours indicates that hyphal development was actively occurring within infected larvae (Fig. S1). Class V myosin is required for hyphal extension, septation, conidiation and conidial germination [35]. In addition, chitin synthase and chitin synthase ChsE (Table S2.7) are essential for hyphal development and were also detected in the proteomic screen. Chitin synthase has been attributed to hyphal development and virulence in murine corneal infection [36]. Analysis of the * G. mellonella* proteomics results identified increased abundance of proteins associated with inflammation at 96 hours, including inter-alpha-trypsin inhibitor heavy chain H4-like isoform X6 and leukotriene A-4 hydrolase-like protein. ITIH4 is linked to cell proliferation, as well as migration during the acute-phase inflammatory response, and serves a role in inflammatory and infectious responses, particularly in bacterial bloodstream infection [37]. Leukotriene A-4 hydrolase-like protein is an important inflammatory modulator, both promoting production of the pro-inflammatory mediator leukotriene B_4_ but also degrading the neutrophil chemoattractant Pro-Gly-Pro (PGP) which could reduce inflammation [38,39]. The production of antimicrobial peptides such as cecropin-d-like peptide and gloverin-like protein- and fungal-specific recognition such as beta-1,3-glucan-binding protein-like isoform X2 was increased, indicating activation of an immune response of * A. fumigatus* proliferation.

Several *A. fumigatus* proteins involved in enzymatic degradation of tissue which may facilitate host colonization and tissue damage were detected. Proteins such as d-lactate dehydrogenase, aldehyde dehydrogenase, beta-glucosidase and trehalase may be involved in metabolizing tissue (Tables S2.5 and S2.7). The major sugar in insect haemolymph is trehalose; therefore, the detection of trehalase indicates the active metabolism of the larval haemolymph carbohydrate (Fig. 3) [40]. Several identified proteins displayed protease activity (ATP-dependent Clp protease, putative, intermembrane space AAA protease IAP-1, calpain-like protease PalBory and intermembrane space AAA protease IAP-1) (Tables S2.1 and S2.6) and could have a role in metabolizing the protein content of insect haemolymph (Fig. 4). Detection of transcriptional activator of proteases prtT (Table S2.1) in the cell-free haemolymph could indicate that proteases are actively involved in the early steps of sublethal fungal colonization. PrtT is integral to the infection processes as it mediates the expression of extracellular proteases that are involved in the penetration of the pulmonary epithelium [41]. Protease-dependent changes to the host cellular actin cytoskeleton can lead to cell peeling and death [42]. The combination of enzymatic and physical disruption to host membranes during tissue invasion has been identified as the causal agent of haemoptysis in aspergillosis patients, typically arising from bronchial blood vessel damage [43]. Analysis of the *G. mellonella* proteome at 96 hours indicated the release of tissue-specific proteins into cell-free haemolymph, indicating tissue damage, which can be a hallmark of *A. fumigatus* infections [44]. These proteins include tropomyosin-1 isoform X1, troponin T, skeletal muscle isoform X1 and myosin heavy chain muscle isoform X10. Troponin T is used as a biomarker in human diseases involving tissue degradation [45]. It has recently been identified that tissue damage may be mediated by immune response dysregulation driven by secondary metabolites produced during hyphal development through mediation of the PacC regulator, which mediates expression of over 250 secreted proteins, including proteases prtT and nonribosomal peptide synthetase GliP, essential for gliotoxin production, of which evidence of expression was evident in our study and that these molecules work synergistically to drive host epithelial damage [44].

In addition, allergens can promote a proinflammatory state, driving tissue remodelling. These include Hsp70 family protein, which was detected in our analysis (Table S2.2), and Hsp70 is a known fungal allergen classified as an IgE reactive cytosolic protein from germinating conidia [46]. Many other allergens may aggravate asthma symptoms and, in combination with the ability of *A. fumigatus* to colonize the airways, could drive the sustained release of allergens [47].

Several pathogenic *Aspergillus* species produce secondary metabolites from aromatic amino acids, including fumiquinazoline and fumitremorgin, which can be derived from tryptophan [48]. Nonribosomal peptide synthetase 13 and tryprostatin B 6-hydroxylase were detected (Table S2.1), and both are involved in fumitremorgin production. This mycotoxin elicits tremorgenic effects resulting in tremors, seizures and abnormal behaviour in mice [49], and fumitremorgin could be responsible for reduced movement of *G. mellonella* larvae (Fig. 2). Nonribosomal peptide synthetase fmqA was also detected and is involved in fumiquinazoline C production, a conidia-associated metabolite with anti-phagocytic properties [50]. *fmqA* is required to produce fumiquinazoline metabolites because it condenses antranilic acid, l-tryptophan and l-alanine in the presence of ATP to form fumiquinazoline C [50].

Many of the above products are derived from aromatic amino acids, which can be synthesized through the Shikimate pathway [51]. The detection of pentafunctional AROM polypeptide (Table S2.1) along with other aromatic amino acid biosynthesis proteins as shown in the STRING analysis (Fig. S2) indicates that this pathway may be involved in the virulence of *A. fumigatus in vivo*. Another detected protein involved in virulence was indoleamine 2,3-dioxygenase subfamily (IDO) (Table S2.1). It is a key enzyme important in immune homeostasis and converts tryptophan to kynurenine and related metabolites. This enzyme is an essential component of host responses to *Aspergillus* and can be exploited by this pathogen as a method of immune evasion [52]. IDO inhibits macrophage recruitment and phagocytosis in *A. fumigatus* keratitis and is involved in M1 macrophage polarization [53].

Gliotoxin is a secondary metabolite implicated in fungal virulence, and the toxicity is attributed to the presence of a disulfide bridge across a piperazine ring. The toxin serves many functions, including oxidative stress homeostasis [54] and suppressing the activity of the NADPH oxidase complex in neutrophils [55] and in insect haemocytes [56]. The secretion of this toxin may suppress the local immune response, enabling the continued persistence of *A. fumigatus*. The detection of gliotoxin is clinically relevant as indicated by numerous studies characterizing its role in immunomodulation [57]. The concentration detected in this study (Fig. 5) at 72 hours (14.2 ± 3.6 µg /larva) was higher than that detected at 72 hours in *A. fumigatus*-infected murine lung tissue (6–8 µg g^−1^) [58,59].

Siderophore concentration also increased over time and reached a level of 12.59 ± 0.70 µg /larva at 96 hours (Fig. 6). Siderophores are small iron-chelating molecules that play an essential role in acquiring iron which is essential for fungal growth. The release of iron from the host through haemolysis can bolster fungal growth and development through acquisition by siderophores [60]. Proteomic analysis detected proteins associated with siderophore production and activity, such as nonribosomal peptide synthetase sidC involved in ferricrocin synthesis [61], and fusarinine C esterase SidJ (Table S2.1), which hydrolyses internalized siderophores [62]. SidC is involved in conidial iron storage required for germ tube formation, asexual sporulation, catalase A activity and virulence [63].

Cell wall-associated proteins in the cell-free haemolymph included chitin synthase, chitin synthase ChsE, alpha 1, 2 mannosidase and conidial pigment polyketide synthase alb 1 (Table S2.1). Conidial pigment polyketide synthase alb1 is associated with the production of DHN-melanin. DHN-melanin disruption results in the formation of smooth conidia and subsequently increases phagocytosis by neutrophils. Deleting alb1 deletion results in a significant loss of virulence in murine models [64].

The wide range of secondary metabolites produced by *A. fumigatus* and biological processes occurring in infected larvae could physically and enzymatically damage tissue, sequester nutrients, alter larval behaviour and suppress the immune response of the host. The results presented here demonstrate that even in the absence of larval death, *A. fumigatus* produces a wide range of potent metabolites and proteins, which have the capacity to damage host tissue or alter the immune response. Prolonged colonization of pulmonary tissue by *A. fumigatus* may lead to the release of similar metabolites that may have adverse effects on the host and may facilitate long-term fungal persistence.

## supplementary material

10.1099/jmm.0.001844 Uncited Supplementary Material 1.

## Abbreviations

ABPA: allergic bronchopulmonary aspergillosis
CPA: chronic pulmonary aspergillosis
IAA: iodoacetamide
