# Supplementary material for: Characterization of Aspergillus fumigatus secretome during sublethal infection of Galleria mellonella larvae
Source: J Med Microbiol. 2024 Jun 5;73(6):001844. doi: 10.1099/jmm.0.001844 (PMC11261830; doi:10.1099/jmm.0.001844)
Supplement: Uncited Supplementary Material 1. [file jmm-73-01844-s001.pdf]

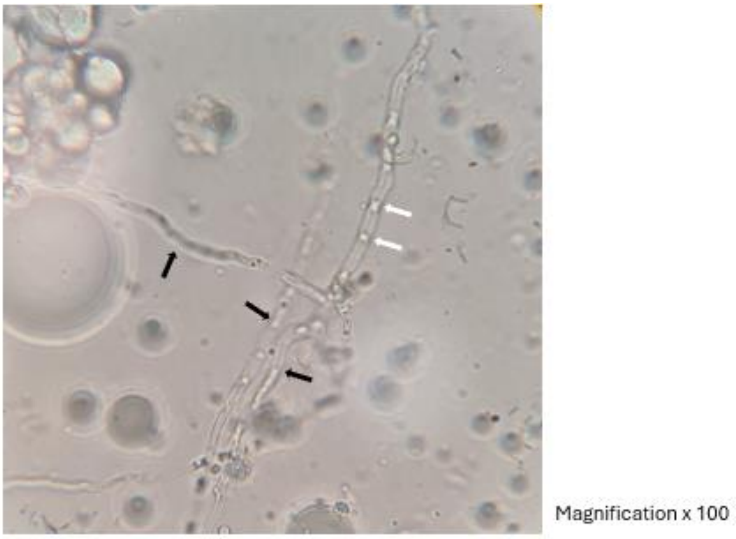

Figure S1: ***A. fumigatus*** **hyphae from 96 hours infected** ***G. mellonella*** **larvae imaged by brightfield microscopy under x100 magnification. Black arrows indicate hypha, white arrows indicate hyphal septa**

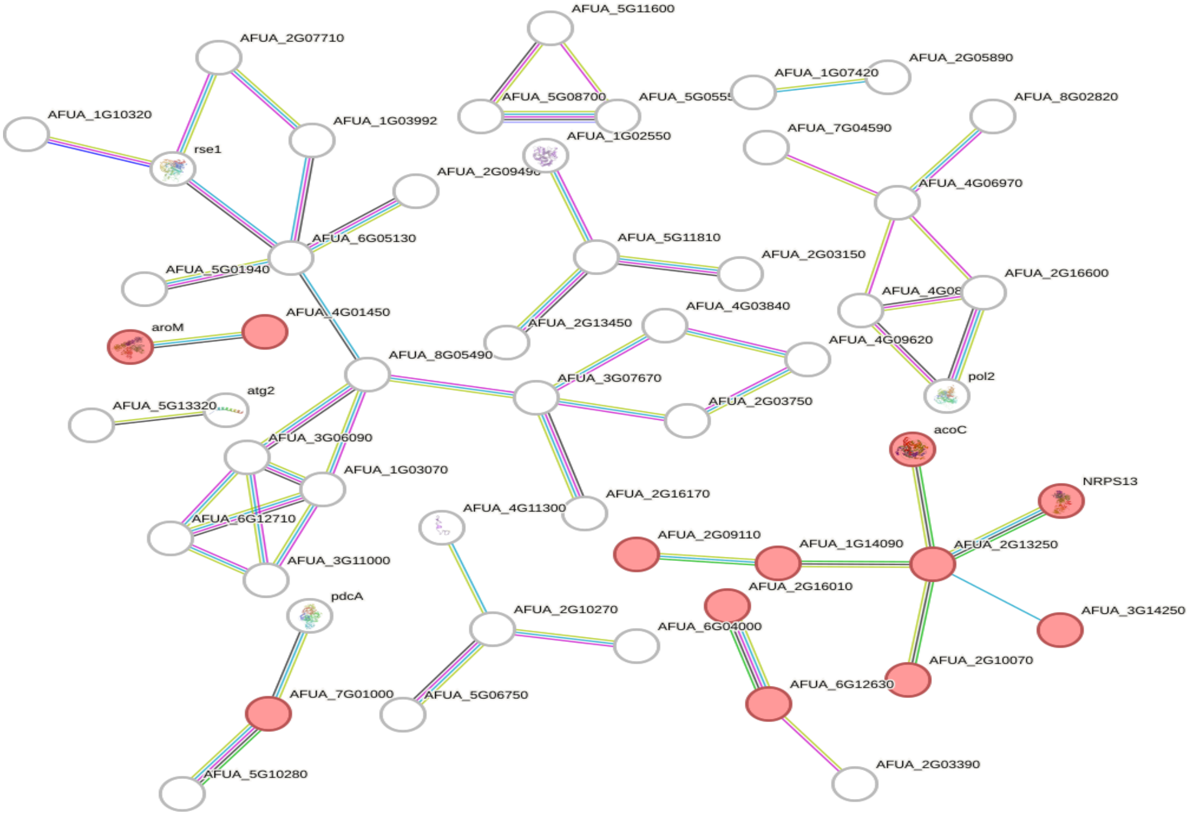

Figure S2: STRING analysis of high confidence ***A. fumigatus*** **protein extracted from 96 hour infected** ***G. mellonella*** **larvae. Proteins associated with aromatic amino acid biosynthesis are indicated in red**

Table S1 *A. fumigatus* proteins detected in control removed from further analysis.

Accession	Description
Q4WRJ7	Uncharacterized protein
Q4WKF2	Small nucleolar ribonucleoprotein complex subunit Dip2, putative
Q4WWZ2	ABC1 domain protein
Q4WX92	Isocitrate dehydrogenase [NADP]
A4D9K5	Ubiquitin-protein ligase Sel1/Ubx2, putative
Q4WWF5	Alpha,alpha-trehalose-phosphate synthase subunit Tps2, putative
Q4WNK4	Uncharacterized protein
Q4X0R9	Uncharacterized protein
Q4WCL1	Protein cft1
Q4WYX5	Fungal specific transcription factor, putative
Q4WI22	Polarized growth protein (Boi2), putative
Q4WDH2	Actin Act1
Q4WW20	NmrA-like family protein
Q4WLE9	C6 finger domain protein, putative
Q4WDH3	Hsp70 nucleotide exchange factor fes1
Q4WXV2	Clustered mitochondria protein homolog
Q4WP94	26S proteasome regulatory subunit RPN2
Q4WFW1	Pfs domain protein
Q4WFC9	Nucleoside-diphosphate-sugar epimerase family protein
Q4WRQ5	C6 transcription factor (UaY), putative
Q4WKG9	RNA-binding protein, putative
Q4WZV6	Xylanolytic transcriptional activator xlnR

Table S2.1 *A. fumigatus* proteins detected associated with Virulence

Accession	Description
Q4WM58	Amino acid permease, putative
Q4WVG9	C2H2 transcription factor, putative
Q4WAE6	calcium transporting ATPase (Pmc1)
Q4WK75	Calpain-like protease PalBory
Q4WR18	Cytochrome P450 monooxygenase helB2
Q4WAZ0	Dual-functional monooxygenase/methyltransferase psoF
Q4WLP8	GPI transamidase component (GAA1), putative
Q4WPB2	GPI-anchored cell surface glycoprotein, putative
Q4WYU0	Indoleamine 2,3-dioxygenase subfamily
Q4WAW3	Nonribosomal peptide synthetase 13
Q4WLW5	Nonribosomal peptide synthetase fmqA
Q4WR82	Nonribosomal peptide synthetase sidC
Q4WF31	MFS siderochrome iron transporter B
Q4WFQ3	Polyketide synthase, putative
Q4WYG2	nonribosomal peptide synthase GliP-like, putative
A0A067Z9B6	O-methyltransferase af390-400
Q4WS76	Pentafunctional AROM polypeptide
Q4WBH9	PKS-like enzyme, putative
Q4WC23	Toxin biosynthesis protein (Tri7), putative
Q4WAW5	Tryprostatin B 6-hydroxylase
Q4WXL9	Vacuolar iron transporter Ccc1, putative
Q4WPQ8	Transcriptional activator of proteases prtT
Q4WZA8	Conidial pigment polyketide synthase alb1
Q4WAZ9	PKS-NRPS hybrid synthetase psoA

Q4WF56	Fusarinine C esterase sidJ

Table S2.2 ***A. fumigatus*** **proteins detected associated with response to stress**

Accession	Description
Q4X006	ABC multidrug transporter A-2
Q4WWW3	ABC multidrug transporter atrI
Q4WFQ4	ABC multidrug transporter H
E9RBG1	ABC multidrug transporter C
Q4WI04	Histidine kinase
Q4WQV0	Glutathione S-transferase, putative
Q4W941	Fatty acid oxygenase, putative
Q4WGE5	Gamma-glutamyltranspeptidase
Q4W9S0	Ferric-chelate reductase, putative
Q6Q487	Calnexin homolog
Q4WYP6	Cation transporting ATPase, putative
Q4WLK5	Autophagy-related protein 2
Q4WRJ0	Copper-transporting ATPase, putative
Q4WE87	Inositol polyphosphate phosphatase, putative
Q4WQQ7	MFS multidrug resistance transporter, putative
Q4WQU4	Hsp70 family protein
Q4WJ30	Molecular chaperone Hsp70
Q4WTE0	PAB1 binding protein (Pbp1), putative
Q4WU06	protein kinase (Gcn2), putative
Q4WYC3	sensor histidine kinase/response regulator, putative
Q4X0F6	Serine/threonin protein kinase, putative
Q4WJH7	Serine/threonine protein kinase, putative
Q4X1E3	Serine/threonine-protein kinase TOR
Q4WPU3	Sphinganine-1-phosphate aldolase BST1, putative
Q4WA71	squalene-hopene-cyclase
A4D9S5	Thermotolerance protein
Q4WLR1	Leucyl-tRNA synthetase
Q4WM74	UV-endonuclease UVE-1
Q4WM37	Bifunctional pyrimidine biosynthesis protein (PyrABCN), putative
Q4WPF2	Serine/threonine-protein kinase atg1
Q4WQ57	alpha-ketoglutarate dehydrogenase complex subunit Kgd1, putative
Q4WWG5	GATA transcription factor LreA
Q4X212	Leashin
Q4WG33	Succinate-semialdehyde dehydrogenase

Table S2.3 ***A. fumigatus*** **proteins detected associated with DNA repair and replication**

Accession	Description
Q4WHA1	Fungal specific transcription factor, putative
Q4WT72	Formin binding protein (FNB3), putative
Q4WTM6	C6 finger domain protein, putative
Q4WHY6	DNA excision repair protein (Rad26L), putative
Q4WIP8	DNA helicase, putative
Q4WP77	DNA mismatch repair protein
Q4WXH8	DNA polymerase epsilon catalytic subunit A

Q4WZK6	DNA polymerase
Q4W9T0	DNA repair protein Rhp26/Rad26, putative
Q4WZQ0	DNA-directed RNA polymerase III RPC4, putative
Q4WA08	DEAD/DEAH box helicase, putative
Q4WI43	Kinesin family protein
Q4WP83	Mediator of RNA polymerase II transcription subunit 8
Q4WT44	Nuclear localization protein, putative
Q4X0H5	Dynactin, putative
Q4WUV0	Nucleoside transporter, putative
Q4WNU3	RAD52 DNA repair protein RADC
Q4X1Q7	SNF2 family helicase, putative
Q4WDL2	snRNA cap binding complex subunit (Gcr3), putative
Q4WL05	SWI/SNF family DNA-dependent ATPase, putative
Q4WGC8	Telomerase reverse transcriptase
Q4WWZ7	Transcription elongation factor S-II
Q4WC71	Transcription initiation factor IIF subunit beta
Q4WWJ4	Transcription initiation factor TFIID subunit 12, putative
Q4WKB3	Transcription initiation factor TFIID subunit, putative
Q4WXX2	Transcriptional activator spt7
Q4X0T9	Kinetochore protein fta7
Q4WPK8	WD repeat protein
Q4WHK4	KAR9-domain-containing protein
Q4WW30	C6 transcription factor, putative
Q4WYJ5	Rad2-like endonuclease, putative
Q4WPD1	Telomere length regulator protein (Rif1), putative
Q4WJG4	Mediator complex subunit 15 KIX domain-containing protein
Q4WTZ1	SNF2 family helicase/ATPase, putative
Q4WVC2	General transcription and DNA repair factor IIH subunit TFB4
Q4WZE7	C2H2 transcription factor (AmdX), putative
Q4WZI5	PHD finger domain protein, putative
Q4X0M8	Mus7/MMS22 family-domain-containing protein
Q4X153	Origin recognition complex subunit 4

Table S2.4 *A. fumigatus* Proteins detected associated with Translation

Accession	Description
Q4X1M0	Eukaryotic translation initiation factor subunit eIF-4F, putative
Q4WMY9	HEAT repeat protein (DRIM), putative
Q4WUC8	Leucine permease transcriptional regulator (SAC3), putative
Q4X247	mRNA splicing factor RNA helicase (Cdc28), putative
Q4WXY3	Nucleolar protein 9
Q4WP57	Elongation factor G, mitochondrial
Q4WLI5	Pre-mRNA-splicing factor rse1
Q4WZR7	Prolyl-tRNA synthetase
Q4W9P0	tRNA-guanosine(34) queuine transglycosylase
Q4WE67	R3H domain protein, putative
Q4WWP3	Ribosomal assembly complex component Ipi3, putative
Q4WHF3	RNA binding protein Jsn1, putative
Q4WQN6	rRNA methyltransferase 1, mitochondrial
Q4WWR4	RNA-dependent RNA polymerase

Q4WYH3	t-complex protein 1, gamma subunit (Cct3)
Q4WES2	tRNA-splicing endonuclease subunit Sen2
Q4X0V6	Midasin
Q4WG50	ARF GTPase activator (Csx2), putative
Q4WKB9	Pre-mRNA-splicing factor cwc22
Q4WP97	mRNA-capping enzyme subunit beta
Q4WHE7	Differentiation regulator (Nrd1), putative
Q4WH96	Mediator of RNA polymerase II transcription subunit 14

Table S2.5 *A. fumigatus* proteins detected associated with metabolism

Accession	Description
Q4WFP9	3-methyl-2-oxobutanoate dehydrogenase, putative
Q4WEK7	Glutamine-dependent NAD(+) synthetase
Q4WDU7	GABA permease, putative
Q4WEE5	phosphoribosylformylglycinamidine synthase
Q4X1G3	Carbamoyl-phosphate synthase, large subunit
Q4WAU8	D-lactate dehydrogenase (Cytochrome)
Q4WJ21	Adenylate kinase
Q4WLR8	AAA family ATPase, putative
Q4WAE3	Aldehyde dehydrogenase, putative
Q4X1M8	Alpha-N-acetylglucosaminidase, putative
Q4WEU9	AMP-binding enzyme, putative
Q4WRY0	Beta-glucosidase, putative
Q4X0G8	Fermentation associated protein (Csf1), putative
Q4WD99	GTPase activating protein (Tsc2), putative
Q4WS41	Imidazole acetol-phosphate transaminase
Q4WQR5	Metal homeostatis protein bsd2
Q4WCY1	Metalloreductase, putative
Q4WS29	Nucleoside-diphosphate-sugar epimerase, putative
Q4WV01	Oxidoreductase, acting on the CH-OH group of donors, NAD or NADP as acceptor
Q4WC62	Oxidoreductase, short chain dehydrogenase/reductase family
Q4WDY2	MFS monosaccharide transporter, putative
Q4X1Q8	Phenylalanine ammonia-lyase
Q4W9G6	Phospho-2-dehydro-3-deoxyheptonate aldolase
Q4X0A5	Probable arabinan endo-1,5-alpha-L-arabinosidase B
Q4WN21	Probable HECT-type ubiquitin ligase-interacting protein creD
Q4WBR0	Putative aconitate hydratase
Q4WUD7	Pyridoxal 5'-phosphate synthase (glutamine hydrolyzing)
Q4WW84	Pyrroline-5-carboxylate reductase
Q4WXX9	Pyruvate decarboxylase
Q4WYW6	Rhamnogalacturonan acetylesterase RgaE
Q4WQP6	Short chain dehydrogenase/reductase family
Q4WMU1	S-methyl-5'-thioadenosine phosphorylase
Q4WGF7	Sterol glucosyltransferase, putative
Q4WZV9	Sulfite reductase, putative
Q4WQP4	Trehalase
Q4X0J6	Tryptophan synthase
Q4WMA5	Ubiquitin-protein ligase (Asi3), putative
Q4WAM5	UTP--glucose-1-phosphate uridylyltransferase

Q4WQ15	Xanthine dehydrogenase
Q4WAN0	Nicotinate phosphoribosyltransferase
Q4WU87	Pyruvate carboxylase, putative
Q4WWC7	Dihydrolipoyllysine-residue succinyltransferase

Table S2.6 *A. fumigatus* proteins detected that are released intracellular proteins

Accession	Description
Q4WHC5	Component of oligomeric Golgi complex 4
Q4WEZ7	Cytochrome P450 monooxygenase, putative
Q4WXN2	Electron-transferring-flavoprotein dehydrogenase
Q4WU40	GRAM domain protein
Q4WND0	alpha-1,2-Mannosidase
A4DA81	Holocytochrome c-type synthase
Q4WQ45	Intermembrane space AAA protease IAP-1
Q4WB84	Mitochondrial carrier protein (Pet8), putative
Q4WVM8	Nuclear pore complex subunit Nup192, putative
Q4WWD2	Mitochondrial tricarboxylate transporter (Ctp), putative
Q4WZL8	Palmitoyltransferase pfa3
Q4WE05	Phosphate permease
Q4WGZ7	Putative allantoate permease of the major facilitator superfamily
Q4WPA2	Regulatory protein Ral2, putative
Q4WC86	Signal peptidase complex subunit 2
Q4WJ32	SNARE complex subunit (Bet1), putative
Q4WQI6	Sorting nexin-4
Q4X1E1	Mitochondrial ribosomal protein MRP51
Q4WWU3	UPF0016 domain protein, putative
Q4WI49	Vacuolar ABC heavy metal transporter (Hmt1), putative
Q4WTX6	Vacuolar protein sorting protein DigA
Q4WBM1	Vacuolar protein sorting/targeting protein 10
Q4WQ23	V-type proton ATPase subunit a
A4D9I4	Putative zinc-finger domain-containing protein
Q4X1U5	ZIP metal ion transporter, putative
Q4WVU2	UV radiation resistance protein (UVRAG), putative
Q4WSQ7	Mitochondrial dynamin GTPase (Msp1), putative
Q4WKK3	ATP-dependent Clp protease, putative

Table S2.7 *A. fumigatus* proteins detected associated with cellular development and cycle

Accession	Description
Q4WCK9	Beta-N-acetylglucosaminidase, putative
Q4WE56	Extracellular endoglucanase, putative
Q4WCK7	Betaine aldehyde dehydrogenase (BadH), putative
Q4WTN8	Class V myosin (Myo4), putative
Q4WBE6	Glycosyltransferase, putative
Q4WTD9	Actin-interacting protein (Bud6/Aip3), putative
Q4WEN2	Cdc48-dependent protein degradation adaptor protein (Shp1), putative
E9QVU7	Cell division control protein Cdc48
Q4WBM5	Cell cycle checkpoint protein Rad17, putative

E9QXP3	Centrin-binding protein Sfi1, putative
Q4X0H6	chitin synthase ChsE
Q4WC58	Chitin synthase
Q4WQ59	Chromatin modification-related protein
Q4WRV5	Gelsolin repeat protein, putative
Q4WD98	GAS2 domain protein
Q4WI19	histone deacetylase RpdA/Rpd3
Q4WDM7	Meiosis protein MEI2, putative
Q4X0D7	Methyltransferase type 11 domain-containing protein
Q4WER2	Mitochondrial fusion protein (Ugo1), putative
Q4WSQ9	Mucin, putative
Q4WUJ7	Myosin type II heavy chain, putative
Q4WAU0	Oxysterol binding protein (Osh1), putative
Q4WVE4	Dynein heavy chain, cytoplasmic
Q4WB96	Pectin methylesterase
Q4WYA3	Phosphatidyl synthase
Q4WG47	phosphatidylinositol 4-kinase (STT4),
Q4WU35	Proteasome regulatory particle subunit Rpt2, putative
Q4WGB4	Rho GTPase activator (Bem2), putative
Q4WWL3	Rho GTPase activator (Rgd1), putative
Q4WKG5	Tubulin alpha chain
Q4WLQ3	Ubiquitin C-terminal hydrolase Ubp8, putative
Q4WK82	UDP-N-acetylglucosaminyltransferase
Q4WRE5	Spt20-like SEP domain-containing protein
Q4WGT0	Glycosyl hydrolase, putative
Q4WX14	Glycosyl transferase, putative
Q4WVN4	Nonribosomal peptide synthetase 8
Q4WKG6	Cytoskeletal adapter protein sagA
Q4WP17	Clathrin heavy chain
Q4X093	Coronin
Q4WVB6	Serine/threonine protein kinase (Nrc-2), putative
Q4WXP6	Ccr4-Not transcription complex subunit (NOT1), putative
Q4WYB3	Guanine nucleotide exchange factor, putative
Q4WGC9	Dynamin-binding protein
Q4WNY0	Ubiquitin fusion degradation protein UfdB, putative
Q4WPX3	HECT-type E3 ubiquitin transferase
Q4WX37	Checkpoint protein kinase, putative
Q4WXS0	Meiotic sister chromatid recombination protein Ish1/Msc1, putative
Q4WT14	Tubulin beta chain
Q4WYC5	Sensor histidine kinase/response regulator, putative
Q4X143	alpha-1,3-glucan synthase Ags2
Q4X245	Nuclear migration protein, putative
Q4WVC4	Actin cytoskeleton organization protein (Cro1), putative
Q4WM86	Carboxylic ester hydrolase

Table S3.1a *G. mellonella* proteins detected in PBS control larvae

**Immune response**	
A0A6J1WD07	27 kDa hemolymph protein

P85216	Anionic antimicrobial peptide 2
A0A6J1WNC9	Apolipophorin-3
P80703	Apolipophorin-3
A0A6J1X726	Apolipophorins isoform X1
A0A6J1X7A0	Apolipophorins isoform X2
A0A6J1W9L8	Apolipoprotein D-like
A0A6J1WMA8	arginine kinase
Q24995	Arylphorin
A0A6J1WVA9	Arylphorin subunit alpha-like
Q0E666	Beta-1,3-glucan-binding protein 1
A0A6J1X785	Beta-1,3-glucan-binding protein-like isoform X1
A0A6J1WHG0	Cathepsin B
A0A6J1W7W7	Cathepsin L
A0A6J3CAU0	Hemocytin-like
A0A6J3C721	Hemocytin-like
C7ASJ3	Hemolin
A0A6J1X1Z8	Leukotriene A-4 hydrolase-like
A0A6J3C047	LOW QUALITY PROTEIN: apolipophorins-like
A0A6J1W7W4	Lysozyme
A0A6J1WR45	Macrophage mannose receptor 1-like isoform X1
A0A6J1X3D5	N-acetylmuramoyl-L-alanine amidase
A0A6J1WTH3	Peptidoglycan recognition protein
A0A6J1W8N1	Phenoloxidase-activating factor 2 (Fragment)
A0A6J1WQ96	Phenoloxidase-activating factor 2
A0A6J3CFY5	Proclotting enzyme-like
A0A6J1WUY6	Scolexin B-like
A0A6J1W790	Serine protease inhibitor 77Ba-like
A0A6J1X3Q4	Soluble interferon alpha/beta receptor
A0A6J3BX75	Spondin-1
A0A6J1W7V7	Transferrin
A0A6J1WGG7	Transgelin
A0A6J1X611	Transgelin
A0A6J1WSG2	tyrosinase
Q964D5	tyrosinase
A0A6J1WMH8	CLIP domain-containing serine protease
A0A6J1X3N4	CLIP domain-containing serine protease
A0A3G1T150	Seroin 3
**Metabolism**	
A0A6J1WLV3	3-ketoacyl-CoA thiolase, mitochondrial-like
A0A3G1T1N8	Acyl-CoA-binding protein homolog
A0A6J1WXC9	aldehyde dehydrogenase (NAD(+))
A0A6J1WLD3	Aldehyde dehydrogenase X, mitochondrial-like
A0A6J1WK19	Beta-galactosidase-like
A0A6J1WWZ0	Beta-hexosaminidase
A0A6J1WFW1	Bile salt-activated lipase-like
A0A6J1WYW8	CAD protein isoform X2
A0A6J1WGS4	Dihydrolipoyllysine-residue succinyltransferase component of 2-oxoglutarate dehydrogenase complex, mitochondrial
A0A6J3C6J9	Dipeptidase
A0A6J3C472	Dipeptidase
A0A6J1X640	Enolase
A0A3G1T1H0	Fatty acid binding protein 1
A0A6J1W9W1	fructose-bisphosphatase
A0A6J1WK99	Fructose-bisphosphate aldolase
A0A6J1WFL2	fumarate hydratase
A0A6J1WMI2	Glyceraldehyde-3-phosphate dehydrogenase

A0A6J1WUC5	Glyceraldehyde-3-phosphate dehydrogenase
A0A6J1WXJ4	Isocitrate dehydrogenase [NADP]
A0A6J1WJ94	L-gulonate 3-dehydrogenase
A0A6J1W9E7	Lipase 3-like
A0A6J3C1Z4	Lysosomal alpha-mannosidase-like
A0A6J3CED7	Malate dehydrogenase
A0A6J1WUN0	Malic enzyme
A0A6J1X3Y0	Multifunctional protein ADE2
A0A6J1WDS4	Pancreatic triacylglycerol lipase-like
A0A6J1X4T9	Probable alpha-mannosidase At5g66150
A0A6J1WYK7	Putative hydroxypyruvate isomerase
A0A6J1WL51	Pyruvate kinase
A0A6J3C2B6	Spermine oxidase-like
A0A6J1X5M2	Succinate--CoA ligase [ADP-forming] subunit beta, mitochondrial
A0A6J1X5I7	transketolase
A0A6J1X7A5	Triosephosphate isomerase
A0A6J3CDF3	UTP--glucose-1-phosphate uridylyltransferase
A0A6J3BT64	Vanin-like protein 2
A0A6J1WVF8	Lipid storage droplets surface-binding protein 1 isoform X1
**cellular structure**	
A0A6J3CA50	N-acetylneuraminate 9- O-acetyltransferase
A0A3G1T170	Actin 3
A0A6J1X2Z5	Actin, muscle
A0A6J1X8F2	Actin-interacting protein 1
A0A6J3BZE6	Allergen Tha p 1-like isoform X2
A0A5C0E4B2	Allergen Tha p 1-like
A0A6J3C2M0	Ankyrin repeat domain-containing protein 13B isoform X2
A0A6J1X888	Cartilage oligomeric matrix protein
A0A6J3CD71	Cadherin-related tumor suppressor
A0A6J1WGF3	Cofilin/actin-depolymerizing factor homolog
A0A6J3BW32	Collagen alpha-1(XI) chain-like isoform X2
A0A6J1X9D6	Endoplasmic reticulum chaperone BiP
A0A6J1WUF0	Enoyl-[acyl-carrier-protein] reductase, mitochondrial
A0A6J1WAR2	Fibrohexamerin
A0A6J1WDH4	Hemicentin-1-like
A0A6J1WK37	Hemicentin-1-like
A0A6J1WWI8	NPC intracellular cholesterol transporter 2
A0A6J1WVR2	Nuclear pore complex protein Nup98-Nup96-like
A0A6J1X6C5	Nucleolar protein dao-5-like
A0A6J1WPL2	Presequence protease, mitochondrial
A0A6J1X7X3	propanoyl-CoA C-acyltransferase
A0A6J1WTL5	V-type proton ATPase subunit a
**insect development**	
A0A6J1WTU8	Alpha-crystallin A chain
A0A6J1X1Q8	Acetylcholinesterase-like isoform X1
A0A6J1WN20	Acidic juvenile hormone-suppressible protein 1
A0A6J1WCF2	Alaserpin-like isoform X5
A0A6J1WD23	Alaserpin-like isoform X7
A0A6J1W9N5	Aldo-keto reductase AKR2E4-like
A0A6J3C2Y3	Aldo-keto reductase AKR2E4-like
A0A6J3CCV7	Aldo-keto reductase AKR2E4-like
A0A6J1WW99	Aldo-keto reductase AKR2E4-like
A0A6J1WSV4	Alpha carbonic anhydrase 8-like
A0A6J3C8E8	Angiotensin-converting enzyme
A0A6J1X4K1	Antichymotrypsin-2-like
A0A6J1WF64	Basic juvenile hormone-suppressible protein 1
A0A6J1WMG8	Basic juvenile hormone-suppressible protein 2-like

A0A5C0E3Y6	Chemosensory protein 14
A0A5C0E3F0	Chemosensory protein 16
A0A6J1W7X0	Juvenile hormone-binding protein
A0A6J1W7P3	Juvenile hormone-binding protein-like
A0A6G6C234	Odorant-binding protein
Q24996	Bilin-binding protein-like
A0A6J1WVW7	Chitinase-like protein EN03 isoform X2
A0A6J3C399	Chymotrypsin-like elastase family member 2A
A0A6J1WKP0	Counting factor associated protein D
A0A6J1WCK9	Cysteine proteinase B-like
A0A6J3CAT5	Extensin-like isoform X1
A0A6J1X4G6	Fibroin heavy chain
A0A6J1WRM7	Inactive serine protease 43 isoform X1
A0A6J1X3A5	Inter-alpha-trypsin inhibitor heavy chain H4-like isoform X5
A0A6J1X1A8	Keratin-associated protein 19-2
A0A6J1WSU8	Lysine-specific demethylase 6A
A0A6J1WFM3	MD-2-related lipid-recognition protein-like
A0A6J3C9T9	Methanethiol oxidase
A0A6J3BYE1	Myotubularin-related protein 13
A0A6J3CCN9	Neuroglian
A0A6J1WPQ2	Possible lysine-specific histone demethylase 1-like
A0A6J3C7J3	Serine protease nudel
A0A6J1WET7	Serpin B8-like
A0A6J1WRD5	Venom carboxylesterase-6-like
A0A6J1WR80	L-dopachrome tautomerase yellow-f2-like
A0A6J1WFY8	Lysosomal aspartic protease
A0A6J1WLM8	Serine protease gd-like
A0A6J1X6G2	Serine protease Hayan-like
**Muscle proteins**	
A0A6J3BRQ3	Myogenesis-regulating glycosidase
A0A6J1X5R5	Cytoplasmic dynein 2 heavy chain 1
A0A6J3C4V9	Dynein heavy chain 5, axonemal isoform X2
A0A6J1WPK4	non-specific serine/threonine protein kinase
A0A3G1T1A8	Thymosin
**Transcription/Translation**	
A0A6J1X2U4	Alpha-N-acetylglucosaminidase
A0A6J1WNG5	Aminoacylase-1-like
A0A6J3C6V1	Aminoacylase-1-like
A0A6J3BVS9	Apyrase-like
A0A6J3BTY5	Centromere-associated protein E
A0A6J1WTX5	Elongation factor 1-alpha
A0A6J1WSG1	Endoribonuclease
A0A6J1WT50	Endoribonuclease
A0A3G1T1K4	Nucleoside diphosphate kinase
A0A6J1X0S1	Polycomb protein suz12-B
A0A6J1X7S0	Peptidyl-prolyl cis-trans isomerase
A0A6J3BUV2	Serologically defined colon cancer antigen 8 homolog isoform X3
A0A6J1X7S2	Sulfhydryl oxidase
**Detoxification**	
A0A6J1WT49	6-pyruvoyl tetrahydrobiopterin synthase
A0A6J1WKT1	Bleomycin hydrolase
A0A6J1WUL1	Carboxylesterase 4A isoform X3
A0A6J1WBZ7	Dipeptidyl peptidase 3
A0A6J1X7Y3	HDHPR
A0A6J1X5C5	Heat shock 70 kDa protein cognate 4
A0A3G1T1A3	Heat shock protein beta-1 isoform X2

A0A6J1X8E0	Insecticyanin-A-like
A0A6J3C787	Superoxide dismutase [Cu-Zn]
A0A6J1WUH6	Glyoxylate reductase/hydroxypyruvate reductase-like
A0A6J1W702	Ommochrome-binding protein-like
A0A6J1WT18	Ommochrome-binding protein-like
A0A6J1X8M7	Ommochrome-binding protein-like
A0A6J1W995	Spermine oxidase-like

Table S3.1b *G. mellonella* Immune proteins detected in PBS control larvae

AMP	
A0A6J1WD07	27 kDa hemolymph protein
P85216	Anionic antimicrobial peptide 2
A0A6J1W7W4	Lysozyme
A0A3G1T150	Seroin 3
**Coagulation**	
A0A6J3CAU0	Hemocytin-like
A0A6J3C721	Hemocytin-like
A0A6J3CFY5	Proclotting enzyme-like
A0A6J1WUY6	Scolexin B-like
**Fungal response**	
Q24995	Arylphorin
A0A6J1WVA9	Arylphorin subunit alpha-like
Q0E666	Beta-1,3-glucan-binding protein 1
A0A6J1X785	Beta-1,3-glucan-binding protein-like isoform X1
**Immune signalling**	
A0A6J1X3Q4	Soluble interferon alpha/beta receptor
A0A6J1WR45	Macrophage mannose receptor 1-like isoform X1
A0A6J1X3D5	N-acetylmuramoyl-L-alanine amidase
A0A6J1WTH3	Peptidoglycan recognition protein
A0A6J1WGG7	Transgelin
A0A6J1X611	Transgelin
A0A6J1WMH8	CLIP domain-containing serine protease
A0A6J1X3N4	CLIP domain-containing serine protease
**Inflammation**	
A0A6J1X1Z8	Leukotriene A-4 hydrolase-like
A0A6J1W7W7	Cathepsin L
A0A6J1WMA8	arginine kinase
**Iron binding**	
A0A6J1W7V7	Transferrin
**Melanisation**	
A0A6J1W790	Serine protease inhibitor 77Ba-like
A0A6J1WSG2	tyrosinase
Q964D5	tyrosinase
A0A6J1W8N1	Phenoloxidase-activating factor 2 (Fragment)
A0A6J1WQ96	Phenoloxidase-activating factor 2

Nutrient reservoir	
A0A6J1WNC9	Apolipophorin-3
P80703	Apolipophorin-3
A0A6J1X726	Apolipophorins isoform X1
A0A6J1X7A0	Apolipophorins isoform X2
A0A6J1W9L8	Apolipoprotein D-like
A0A6J1WHG0	Cathepsin B
A0A6J3C047	LOW QUALITY PROTEIN: apolipophorins-like
**Pathogen Binding**	
C7ASJ3	Hemolin
A0A6J3BX75	Spondin-1

Table S3.2a *G. mellonella* proteins detected in 96 hour infected larvae

Immune response	
A0A6J1WD07	27 kDa hemolymph protein
P85216	Anionic antimicrobial peptide 2
A0A6J1WNC9	Apolipophorin-3
P80703	Apolipophorin-3
A0A6J1X726	Apolipophorins isoform X1
A0A6J1X7A0	Apolipophorins isoform X2
A0A6J1W9L8	Apolipoprotein D-like
A0A6J1WMA8	arginine kinase
Q24995	Arylphorin
A0A6J1WVA9	Arylphorin subunit alpha-like
A0A6J1WMZ6	Arylphorin subunit alpha-like
Q0E666	Beta-1,3-glucan-binding protein 1
A0A6J1WC08	Beta-1,3-glucan-binding protein-like isoform X2
A0A6J1WV28	Cadherin-99C
A0A3G1T1R1	Calreticulin
A0A6J1W7W7	Cathepsin L
P85210	Cecropin-D-like peptide
A0A6J3BUX9	Defense protein Hdd11
A0A6J1WQI3	Gloverin-like
A0A6J1X834	Hemocyte protein-glutamine gamma-glutamyltransferase-like
A0A6J3C721	Hemocytin-like
C7ASJ3	Hemolin
A0A6J1X2Z4	Inter-alpha-trypsin inhibitor heavy chain H4-like isoform X6
C9WHZ7	Lebocin-4
A0A6J1X1F5	Leukotriene A-4 hydrolase isoform X2
A0A6J1X1Z8	Leukotriene A-4 hydrolase-like
A0A6J3C047	LOW QUALITY PROTEIN: apolipophorins-like
A0A6J1X3D5	N-acetylmuramoyl-L-alanine amidase
A0A6J3C5W3	Neuroglian-like
A0A6J1WTH3	Peptidoglycan recognition protein
A0A6J1W8N1	Phenoloxidase-activating factor 2 (Fragment)
A0A6J1WBF9	Phosphatidylethanolamine-binding protein 1-like
A0A6J1WUY6	Scolexin B-like
A0A6J1W790	Serine protease inhibitor 77Ba-like
A0A3G1T150	Seroin 3
A0A6J1X0P3	Seroin isoform X2
A0A6J3BX75	Spondin-1

A0A6J1W7V7	Transferrin
A0A6J3C158	Transferrin-like isoform X1
A0A6J3BY59	Transferrin-like
A0A6J1WSG2	tyrosinase
Q964D5	tyrosinase
A0A6J3CB97	5-hydroxytryptamine receptor 2C-like isoform X4
A0A6J1X8K1	Mucin-5AC
A0A6J1WNH2	Mucin-5AC-like
A0A6J3C4B5	Ninjurin-1 isoform X3
A0A6J1X347	Plastin-2
A0A6J1X8N5	Serine protease inhibitor 88Ea-like
A0A6J1W8W0	Thymosin beta isoform X1
A0A3G1T1A8	Thymosin
A0A6J1WGG7	Transgelin
A0A6J1X611	Transgelin
**Metabolism**	
A0A6J1WCC6	2-oxoglutarate dehydrogenase, mitochondrial
A0A6J1WUQ0	3-hydroxyacyl-CoA dehydrogenase
A0A6J1WLV3	3-ketoacyl-CoA thiolase, mitochondrial-like
A0A6J1WP78	6-phosphogluconate dehydrogenase, decarboxylating
A0A6J1WQP5	Acetyl-CoA hydrolase
A0A6J3BZ19	Adenosine kinase
A0A6J1WPY4	Adenosylhomocysteinase
A0A6J3CDB2	Adenylate kinase
A0A6J1X0E4	alanine transaminase
A0A6J1WEP3	Aldehyde dehydrogenase X, mitochondrial-like
A0A6J1X345	Argininosuccinate synthase
A0A6J1WQ25	Aromatic-L-amino-acid decarboxylase
A0A6J1WPG6	Aspartate aminotransferase
A0A6J3BYL4	ATP citrate synthase
A0A6J1WK19	Beta-galactosidase-like
A0A6J1WWZ0	Beta-hexosaminidase
A0A6J3BRA4	Beta-ureidopropionase
A0A6J1WSJ6	Bifunctional purine biosynthesis protein ATIC
A0A6J1WFW1	Bile salt-activated lipase-like
A0A6J1WYW8	CAD protein isoform X2
A0A6J1W772	Chitinase-3-like protein 1
A0A6J3C399	Chymotrypsin-like elastase family member 2A
A0A6J1X7E7	Citrate synthase
A0A6J1WEK7	Cubilin
A0A6J3C9I6	cystathionine gamma-lyase
A0A6J1WNL5	Cytoplasmic aconitate hydratase-like
A0A6J1X4Z2	D-3-phosphoglycerate dehydrogenase
A0A6J1WRJ0	Egalitarian protein homolog
A0A6J1X640	Enolase
A0A6J1WWS2	enoyl-CoA hydratase
A0A6J1WSQ5	Enoyl-CoA hydratase, mitochondrial
A0A6J1X026	Facilitated trehalose transporter Tret1-like
A0A3G1T1H0	Fatty acid binding protein 1
A0A6J1WR51	FGGY carbohydrate kinase domain-containing protein
A0A6J1W9W1	fructose-bisphosphatase
A0A6J1WJM9	fructose-bisphosphate aldolase
A0A6J1WK99	Fructose-bisphosphate aldolase
A0A6J1WFL2	fumarate hydratase
A0A6J1WMW3	Fumarylacetoacetase
A0A6J3C3C3	Galectin
A0A6J3C058	Glucose dehydrogenase [FAD, quinone]-like

A0A6J1WB68	Glucose-6-phosphate isomerase
A0A6J1WUD8	glutamate dehydrogenase [NAD(P)(+)]
A0A6J1X8P2	Glutamate--cysteine ligase
A0A6J1WZ44	Glutamine synthetase
A0A6J1WMI2	Glyceraldehyde-3-phosphate dehydrogenase
A0A6J1WUC5	Glyceraldehyde-3-phosphate dehydrogenase
A0A6J1WNU9	Glycerophosphocholine phosphodiesterase GPCPD1 isoform X1
A0A6J3CBL1	glycerophosphodiester phosphodiesterase
A0A6J3C371	glycine hydroxymethyltransferase
A0A6J1WTY4	glycogenin glucosyltransferase
A0A6J1WG11	Inositol-1-monophosphatase
A0A6J1WXJ4	Isocitrate dehydrogenase [NADP]
A0A6J1X9A8	Isocitrate dehydrogenase [NADP]
A0A6J3BYB5	Lactase-phlorizin hydrolase-like
A0A6J1WJ94	L-gulonate 3-dehydrogenase
A0A6J1W9E7	Lipase 3-like
A0A6J1WAL9	Lipase 3-like
A0A6J1WG45	Lipase member I-like
A0A6J3BQW9	Lipoprotein lipase-like
A0A6J1X315	L-lactate dehydrogenase
A0A6J1WPP0	L-xylulose reductase-like
A0A6J3CED7	Malate dehydrogenase
A0A6J1WS44	Malate dehydrogenase, mitochondrial
A0A6J1X2P6	Malate dehydrogenase, mitochondrial-like
A0A6J1WUN0	Malic enzyme
A0A6J1WZK5	Mucin-2 isoform X1
A0A6J1X414	Multifunctional fusion protein
A0A6J1X3Y0	Multifunctional protein ADE2
A0A6J1X3S6	N-acetylneuraminate lyase
A0A6J1WLG4	N-acyl-aliphatic-L-amino acid amidohydrolase
A0A6J3BQN0	Nuclear factor of activated T-cells 5 isoform X5
A0A6J1WZX5	Ornithine aminotransferase
A0A6J1WD77	Pancreatic lipase-related protein 2-like
A0A6J1WZM7	Pancreatic triacylglycerol lipase-like
A0A6J1WUV9	phosphoglucomutase (alpha-D-glucose-1,6-bisphosphate-dependent)
A0A6J1X4N3	Phosphoglycerate kinase
A0A6J1WKC4	Phosphoglycerate mutase
A0A6J3C7Z1	phosphoribosylformylglycinamidine synthase
A0A3G1T1N2	Phosphoserine aminotransferase
A0A6J3C208	Phosphotransferase
A0A6J1WQY1	Probable chitinase 10
A0A6J1WN53	Probable methylmalonate-semialdehyde/malonate-semialdehyde dehydrogenase [acylating], mitochondrial
A0A6J1W7W3	Probable methylthioribulose-1-phosphate dehydratase
A0A6J1X0J3	Purine nucleoside phosphorylase
A0A6J1WYK7	Putative hydroxypyruvate isomerase
A0A6J1WZF9	Pyrimidodiazepine synthase-like
A0A6J1WL51	Pyruvate kinase
A0A6J3C794	Pyruvate kinase
A0A6J1WU94	RING finger protein 17
A0A6J1WV91	Serine/threonine-protein kinase Tor
A0A6J1WYN9	Succinate--CoA ligase [ADP/GDP-forming] subunit alpha, mitochondrial
A0A6J1WZW5	Trans-1,2-dihydrobenzene-1,2-diol dehydrogenase
A0A6J1X5I7	transketolase
A0A6J3C3N6	Trehalase
A0A6J1X7A5	Triosephosphate isomerase
A0A6J3CEC1	Uridine phosphorylase 1 isoform X4

A0A6J1WNV9	Uridine phosphorylase 1-like
A0A6J3CDF3	UTP--glucose-1-phosphate uridylyltransferase
A0A6J1X4M4	UTP--glucose-1-phosphate uridylyltransferase
A0A6J3BT64	Vanin-like protein 2
A0A6J1WLJ3	xanthine dehydrogenase
A0A6J1WHI1	Aconitate hydratase, mitochondrial
A0A6J1WXC9	aldehyde dehydrogenase (NAD(+))
A0A6J1WLD3	Aldehyde dehydrogenase X, mitochondrial-like
A0A6J3BXS6	Apyrase-like
A0A6J1X7X8	ATP synthase subunit alpha
A0A6J1WTH0	ATP synthase subunit beta
A0A6J3BVK5	ATP synthase subunit
A0A6J1WW25	C-1-tetrahydrofolate synthase, cytoplasmic
A0A6J3BV24	Diacylglycerol kinase
A0A6J1WZV6	Electron transfer flavoprotein subunit alpha
A0A6J1W7S8	Hydroxylysine kinase
A0A6J1WMU9	Indole-3-acetaldehyde oxidase-like
A0A6J1WGQ6	inorganic diphosphatase
A0A6J1WWZ3	Long-chain-fatty-acid--CoA ligase
A0A6J1X7Q9	vesicle-fusing ATPase
A0A6J1WES2	Alpha-1,4 glucan phosphorylase
**Cellular structure**	
A0A6J1X9Q1	Laminin subunit beta-1
A0A6J3C6G3	Laminin subunit gamma-1
A0A6J1WF72	Laminin-like protein epi-1
A0A3G1T170	Actin 3
A0A6J1X8F2	Actin-interacting protein 1
A0A3G1T1N8	Acyl-CoA-binding protein homolog
A0A6J1WP12	Acyl-CoA-binding protein homolog
A0A6J1X1D9	Adenylyl cyclase-associated protein
A0A6J3BZE6	Allergen Tha p 1-like isoform X2
A0A6J1WKK0	Aminopeptidase N-like isoform X2
A0A6J1X177	Aminopeptidase
A0A6J1WYY4	Aminopeptidase W07G4.4
A0A6J1WWH2	Ankycorbin isoform X1
A0A6J1WXW1	Annexin
A0A6J1X252	ATPase ASNA1 homolog
A0A6J1WVA0	ATPase H(+)-transporting accessory protein 2
A0A6J3C6I5	Beta-secretase 1-like
A0A6J1X3B9	Calcineurin-binding protein cabin-1-like
A0A6J3C3M6	Calsyntenin-1
A0A6J1W933	Carboxypeptidase Q
A0A6J1X888	Cartilage oligomeric matrix protein
A0A6J3C1C5	Clustered mitochondria protein homolog
A0A6J1WGF3	Cofilin/actin-depolymerizing factor homolog
A0A6J3CDJ4	Collagen alpha-1(I) chain-like isoform X2
A0A6J3BU81	Collagen alpha-1(I) chain-like
A0A6J1WWS9	Collagen alpha-1(IV) chain
A0A6J1X171	Collagen alpha-1(XI) chain-like
A0A6J3CAN1	Constitutive coactivator of PPAR-gamma-like protein 1
A0A6J1WV07	Cuticlin-1
A0A6J1WCK9	Cysteine proteinase B-like
A0A3G1T1H8	Cytochrome b5-like
A0A6J1WU81	Cytochrome c
A0A6J3C513	E1 ubiquitin-activating enzyme
A0A6J1X903	E3 ubiquitin-protein ligase
A0A6J1X9D6	Endoplasmic reticulum chaperone BiP

A0A6J1WHD7	Endoplasmin
A0A6J3C4Z4	F-actin-uncapping protein LRRC16A isoform X2
A0A6J1WAR2	Fibrohexamerin
A0A6J1W907	Gelsolin-like
A0A6J1WG12	H(+)-transporting two-sector ATPase
A0A6J1X5A4	HECT-type E3 ubiquitin transferase
A0A6J1WDH4	Hemicentin-1-like
A0A6J1WK37	Hemicentin-1-like
A0A5C0E3I9	Ionotropic receptor 25a
A0A6J3C198	Kalirin-like isoform X3
A0A6J1X1A8	Keratin-associated protein 19-2
A0A6J1X3E8	Mannosyltransferase
A0A6J1WZ89	Membrane alanyl aminopeptidase-like
A0A6J1X228	Microtubule-associated protein RP/EB family member 1 isoform X1
A0A6J1WYP3	Mitochondrial import inner membrane translocase subunit Tim16
A0A6J3CA50	N-acetylneuraminate 9-O-acetyltransferase
A0A6J1X310	Nidogen
A0A6J3CE91	non-specific serine/threonine protein kinase
A0A6J3C652	non-specific serine/threonine protein kinase
A0A6J1WVR2	Nuclear pore complex protein Nup98-Nup96-like
A0A6J1WIK7	Nucleolar protein 4 isoform X3
A0A6J1WZR9	palmitoyl-protein hydrolase
A0A6J3CBN2	Papilin-like
A0A6J1X8D2	Peroxisomal multifunctional enzyme type 2-like
A0A6J1WHI6	Polyubiquitin-B isoform X2
A0A6J3C0T2	Probable E3 ubiquitin-protein ligase HERC4 isoform X4
A0A6J1WW74	Profilin
A0A6J1WV18	Programmed cell death 6-interacting protein
A0A6J1X117	Programmed cell death protein 5
A0A6J3C8F0	Proteasomal ATPase-associated factor 1-like isoform X2
A0A6J3CDC6	Proteasomal ubiquitin receptor ADRM1 isoform X3
A0A6J1WF39	Proteasome subunit alpha type
A0A6J1X075	Proteasome subunit alpha type
A0A3G1T1M6	Proteasome subunit alpha type
A0A6J1WIF1	Proteasome subunit beta
A0A6J3CDX3	Protein arginine N-methyltransferase 9-like
A0A6J1WS96	Protein DDI1 homolog 2
A0A6J1WWY4	Protein SEC13 homolog
A0A6J1X3H5	Protein transport protein SEC23
A0A6J3C4R5	RCR-type E3 ubiquitin transferase
A0A6J3C332	Regucalcin
A0A6J3C570	Sodium channel protein Nach-like
A0A6J1WWR0	Sodium/potassium-transporting ATPase subunit alpha
A0A6J3C588	Sortilin-related receptor-like
A0A6J1X576	Spectrin alpha chain-like
A0A6J1WVF7	Sterol carrier protein 2
A0A6J1WRZ3	Sterol regulatory element-binding protein cleavage-activating protein
A0A6J1X7S2	Sulfhydryl oxidase
A0A6J1WR37	Tectonin beta-propeller repeat-containing protein
A0A6J1X7A4	TOM1-like protein 2 isoform X4
A0A6J1WT77	Trafficking protein particle complex subunit 8
A0A3G1T187	Tubulin alpha chain
A0A6J1WHZ4	Tubulin beta chain
A0A6J1X0Z9	Tubulin polymerization-promoting protein homolog
A0A6J1X044	Tumor protein D54 isoform X4
A0A6J1WQ35	Vacuolar proton pump subunit B
A0A6J1WIJ6	Vesicular integral-membrane protein VIP36
A0A6J3C3V8	V-type proton ATPase subunit C

A0A6J1X5F4	V-type proton ATPase subunit E
A0A6J1WI64	V-type proton ATPase subunit G
A0A6J1W9F8	V-type proton ATPase subunit S1
A0A6J1X3Q0	Zinc carboxypeptidase-like
**Insect development**	
A0A6J1X073	14 kDa phosphohistidine phosphatase-like isoform X1
A0A6J1WNS7	Abnormal cell migration protein 10 isoform X2
A0A6J1WN20	Acidic juvenile hormone-suppressible protein 1
A0A6J3C0S7	Active breakpoint cluster region-related protein
A0A6J1X2U4	Alpha-N-acetylglucosaminidase
A0A6J1WH15	Adhesion G protein-coupled receptor A3
A0A6J1WCF2	Alaserpin-like isoform X5
A0A6J1WD23	Alaserpin-like isoform X7
A0A6J3BZJ8	Aldehyde oxidase 3-like
A0A6J3C2Y3	Aldo-keto reductase AKR2E4-like
A0A6J3CCV7	Aldo-keto reductase AKR2E4-like
A0A6J1WTU8	Alpha-crystallin A chain
A0A6J3BTR4	Alsin
A0A6J3C8E8	Angiotensin-converting enzyme
A0A6J1WCF7	Antichymotrypsin-2-like isoform X8
A0A6J1WIX3	Antichymotrypsin-2-like isoform X9
A0A6J1X4K1	Antichymotrypsin-2-like
A0A6J1WF64	Basic juvenile hormone-suppressible protein 1
A0A6J1WMG8	Basic juvenile hormone-suppressible protein 2-like
A0A6J3C7E6	Cadherin-23-like
A0A6J3CD71	Cadherin-related tumor suppressor
A0A6J1WM51	Chaoptin
A0A5C0E3Y6	Chemosensory protein 14
A0A5C0E3F0	Chemosensory protein 16
A0A6J1WVW7	Chitinase-like protein EN03 isoform X2
A0A6J1WDE7	Cholinephosphotransferase 1 isoform X2
A0A6J1WC07	Death-associated protein 1
A0A6J3BY29	Dentin sialophosphoprotein-like
A0A6J3C831	Down syndrome cell adhesion molecule-like protein Dscam2
A0A6J1X818	Elastin-like
A0A6J1WNE6	Epoxide hydrolase
A0A6J3C7J1	Fatty acid synthase
A0A6J1X4G6	Fibroin heavy chain
Q26427	Fibroin light chain
A0A6J3C100	Gamma-aminobutyric acid receptor subunit beta-like isoform X3
A0A6J1WJM8	Gephyrin
A0A6J1WQZ4	Glyoxalase domain-containing protein 4
A0A6J1WST6	GPI ethanolamine phosphate transferase 1
A0A6J3C3F2	Heat shock protein 68-like
A0A3G1T1C6	Heat shock protein 83
A0A6J3C6C6	Inositol hexakisphosphate and diphosphoinositol-pentakisphosphate kinase
A0A6J1X8G5	Integrin alpha-IIb-like
A0A6J1WRV1	Juvenile hormone esterase-like
A0A6J1W7X0	Juvenile hormone-binding protein
A0A6J1WYQ3	Larval cuticle protein A2B-like
A0A6J1WVI3	Luciferin 4-monooxygenase
A0A6J1WGW2	Luciferin 4-monooxygenase-like
A0A6J1WFP0	Male-enhanced antigen 1
A0A6J1WJ72	Mitochondrial glycine transporter
A0A6J3C662	Moesin/ezrin/radixin homolog 1-like
A0A6J1WAP1	Nephrin-like isoform X2

A0A6J3C0K8	Neprilysin-4-like isoform X1
A0A6J3CHW4	Nesprin-1
A0A6J3BWB1	Neurofibromin
A0A6G6C234	Odorant-binding protein
A0A6J1X7S0	Peptidyl-prolyl cis-trans isomerase
A0A3G1T1D0	Peptidyl-prolyl cis-trans isomerase
A0A6J1WFC9	phosphoinositide 5-phosphatase
A0A6J1WF21	Probable salivary secreted peptide
A0A6J1X0G8	Prolyl endopeptidase
A0A6J1WMI4	Protein bark beetle
A0A6J3CFF3	Protein capicua homolog isoform X2
A0A6J1WWJ8	Protein distal antenna-like isoform X2
A0A6J3C4B8	Protein furry-like
A0A6J1WMK0	Protein gustavus isoform X1
A0A6J3C3N4	Protein hu-li tai shao isoform X8
A0A6J1WW42	Protein lethal(2)essential for life-like
A0A6J3C8M5	Protein purity of essence-like
A0A6J1WJP6	protein-tyrosine-phosphatase
A0A6J1X799	Pyrimidodiazepine synthase-like
A0A6J1X9L7	Rab GDP dissociation inhibitor
A0A6J3BP97	Ral GTPase-activating protein subunit beta
A0A6J3C167	Rap1 GTPase-activating protein 1 isoform X4
A0A6J3C8P3	Regulating synaptic membrane exocytosis protein 1 isoform X5
A0A6J1WEK4	Retinal dehydrogenase 1-like
A0A6J3BYE2	Rho GTPase-activating protein 190 isoform X2
A0A6J1X6T9	Rho guanine nucleotide exchange factor 10 isoform X3
A0A6J1WNR7	Rho guanine nucleotide exchange factor 17
A0A6J1WIS6	Serine/threonine-protein phosphatase PP2A 65 kDa regulatory subunit
A0A6J1WYD1	Small glutamine-rich tetratricopeptide repeat-containing protein alpha-like
A0A6J1WN78	Sperm flagellar protein 2-like
A0A6J1WT79	Sushi, von Willebrand factor type A, EGF and pentraxin domain-containing protein 1 isoform X1
A0A6J3CCB9	Synapsin
A0A6J1WY50	Transcription factor Sp9 isoform X2
A0A6J1WEM6	Tyrosine-protein kinase receptor
A0A6J3BZE1	Tyrosine-protein kinase receptor torso-like isoform X1
A0A6J1WTY1	Zinc finger protein 423 homolog
A0A6J1WLM8	Serine protease gd-like
A0A6J1WRR7	15-hydroxyprostaglandin dehydrogenase [NAD(+)]
A0A6J1WH84	15-hydroxyprostaglandin dehydrogenase [NAD(+)]-like
**Muscle proteins**	
A0A6J1X2Z5	Actin, muscle
A0A6J1WGN4	Alpha-actinin, sarcomeric isoform X2
A0A6J1X830	Dynein axonemal assembly factor 1 homolog
A0A6J3CAQ0	Dynein beta chain, ciliary
A0A6J1X455	Dynein beta chain, ciliary-like
A0A6J3C0H7	Dynein heavy chain 10, axonemal-like
A0A6J1X234	Dynein heavy chain 12, axonemal
A0A6J3BR13	Dynein heavy chain 2, axonemal
A0A6J3BSG3	Dynein heavy chain 2, axonemal-like
A0A6J3C4V9	Dynein heavy chain 5, axonemal isoform X2
A0A6J3BQS2	Dynein heavy chain 7, axonemal
A0A6J1WU42	Dynein heavy chain 8, axonemal
A0A6J1X3J9	Dynein light chain
A0A6J3C3Q0	Filamin-A
A0A6J3C622	Kinesin-like protein KIF21B isoform X2

A0A6J1WM33	Myophilin
A0A6J1WLL6	Myophilin-like
A0A6J1X4K6	Myosin heavy chain, muscle isoform X10
A0A6J3BY13	Myosin heavy chain, non-muscle-like
A0A6J3CGX9	Myosin light chain alkali
A0A6J1WPS1	Myosin regulatory light chain 2
A0A6J1WF33	Myosin-2 essential light chain isoform X1
A0A6J1WM80	Myotrophin-like
A0A6J3C606	Obscurin
A0A6J1X0B8	Paramyosin, long form
A0A6J1X247	Protein bicaudal D isoform X1
A0A6J1WKJ2	Protein unc-119 homolog
A0A6J1W7Q4	Protein unc-13 homolog 4B isoform X3
A0A6J3BW05	Ryanodine receptor isoform X17
A0A6J1WUG0	Titin
A0A6J3C0D8	Titin
A0A6J1WXN1	Titin-like
A0A6J3C572	Titin-like
A0A6J1W9N2	Tropomyosin-1 isoform X1
A0A6J1WA54	Tropomyosin-2 isoform X8
A0A6J1WQD6	Troponin C-like
A0A6J1WEM3	Troponin I isoform X11
A0A6J3BU54	Troponin T, skeletal muscle isoform X1
A0A6J3C3G9	Twitchin isoform X3
**Transcription/Translation**	
A0A6J3C3N0	[histone H3]-dimethyl-L-lysine(36) demethylase
A0A3G1T1P6	40S ribosomal protein S12
A0A3G1T1L4	60S ribosomal protein L21
A0A6J1X700	60S ribosomal protein L4
A0A3G1T1E4	60S ribosomal protein L7
A0A6J1X9I8	97 kDa heat shock protein isoform X2
A0A6J1WBJ2	Actin-related protein 8
A0A6J1WWK8	Adenylosuccinate synthetase
A0A6J1WQ04	Alanine--tRNA ligase
A0A6J3C6V1	Aminoacylase-1-like
A0A6J3BSQ2	Ankyrin repeat domain-containing protein 17
A0A6J1WIL2	Ankyrin-3-like isoform X1
A0A6J1WT06	ArfGAP with SH3 domain, ANK repeat and PH domain-containing protein isoform X2
A0A6J1W8P7	ATP-dependent RNA helicase
A0A6J1WVR0	CCR4-NOT transcription complex subunit 1
A0A6J3BTY5	Centromere-associated protein E
A0A6J1WS84	Condensin complex subunit 3
A0A6J1W7T7	Cytosolic purine 5'-nucleotidase isoform X3
A0A6J3BXH5	DNA helicase
A0A6J1X3Q5	DNA polymerase alpha subunit B
A0A6J1WL43	DNA polymerase
A0A6J1X4B1	DNA replication licensing factor MCM5
A0A6J1X0M5	Double-stranded RNA-binding protein Staufen homolog 2-like isoform X1
A0A6J1WTX5	Elongation factor 1-alpha
A0A3G1T1F4	Elongation factor 1-beta'
A0A3G1T1I4	Elongation factor 1-gamma
A0A6J1WSG1	Endoribonuclease
A0A6J1WT50	Endoribonuclease
A0A6J1WWZ2	Enhancer of mRNA-decapping protein 4-like
A0A6J1W8U6	Eukaryotic peptide chain release factor subunit 1
A0A6J3C4X7	Eukaryotic translation initiation factor 4 gamma 3-like isoform X7

A0A6J1WK04	Fanconi anemia group J protein homolog isoform X1
A0A6J1X425	Helicase SKI2W
A0A6J3BTU2	Heterogeneous nuclear ribonucleoprotein 27C
A0A6J1WMW0	Histidine triad nucleotide-binding protein 1-like
A0A6J1WTZ5	Homeobox protein 2-like
A0A3G1T1L2	Large ribosomal subunit protein P2
A0A3G1T175	Large ribosomal subunit protein uL1
A0A3G1T1I3	Large ribosomal subunit protein uL22
A0A6J1X6N2	Mediator of RNA polymerase II transcription subunit 1
A0A6J1WKS2	Methionine aminopeptidase 2
A0A6J1WKW8	Mitotic checkpoint serine/threonine-protein kinase BUB1 beta-like
A0A6J1X0E3	non-specific serine/threonine protein kinase
A0A6J1WKY9	N-terminal acetyltransferase B complex subunit MDM20 homolog
A0A6J1X6C5	Nucleolar protein dao-5-like
A0A3G1T1K4	Nucleoside diphosphate kinase
A0A6J1WEV4	Origin recognition complex subunit 5
A0A6J1WAZ5	peptidylprolyl isomerase
A0A6J1WCV8	Polypyrimidine tract-binding protein 1 isoform X8
A0A6J1WPQ2	Possible lysine-specific histone demethylase 1-like
A0A6J1WLJ5	Probable phospholipid-transporting ATPase IF
A0A6J1WPW5	Probable RNA-binding protein 46
A0A6J1X6K5	Probable serine/threonine-protein kinase fhkE
A0A6J1W9Q2	Proteasome subunit beta
A0A6J3BYD7	Protein mini spindles
A0A6J3BZ59	Protein PFF0380w-like isoform X2
A0A6J1WWF9	RNA helicase aquarius
A0A3G1T185	RNA helicase
A0A6J3CA14	RNA helicase
A0A6J1WXG1	RNA helicase
A0A6J1X2P0	RNA-binding protein squid isoform X2
A0A6J3C8D4	RNA-directed DNA polymerase
A0A6J1WCB5	RuvB-like helicase
A0A6J1WNH6	Scm-like with four MBT domains protein 2
A0A6J1X0Y0	serine--tRNA ligase
A0A6J1WX17	Small nuclear ribonucleoprotein-associated protein B
A0A3G1T1C1	Small ribosomal subunit protein RACK1
A0A3G1T1D1	Small ribosomal subunit protein uS2
A0A6J1WQC1	Splicing factor 3B subunit 1
A0A6J1WEL0	T-complex protein 1 subunit theta
A0A6J1WQK5	Telomere-associated protein RIF1
A0A6J1WI88	Transcription factor BTF3
A0A6J3BQW6	Transcription-associated protein 1
A0A6J1WGR6	Tyrosine--tRNA ligase
A0A6J1WWG2	Zinc finger FYVE domain-containing protein 26 homolog
A0A6J1W8Z1	Zinc finger protein 879-like isoform X4
A0A6J1W827	Adenylate kinase isoenzyme 1 isoform X1
A0A6J1X7W2	Cysteine--tRNA ligase, cytoplasmic
A0A3G1T1H9	GTP-binding nuclear protein
A0A6J3CB71	Helicase domino-like
A0A6J1WT72	Protein disulfide-isomerase A6 homolog
A0A6J1X7G6	Protein disulfide-isomerase
A0A3G1T1E0	Large ribosomal subunit protein uL18
A0A6J1WQ92	Small subunit processome component 20 homolog
A0A6J1WVI0	Staphylococcal nuclease domain-containing protein
A0A6J1WTW7	T-complex protein 1 subunit epsilon
A0A6J1WSA6	T-complex protein 1 subunit zeta
A0A3G1T184	Translation elongation factor 2
A0A3G1T1E2	Translationally-controlled tumor protein homolog

A0A6J1X3X8	Tubulin--tyrosine ligase-like protein 12
**Detoxification**	
A0A6J3BW14	14-3-3 protein epsilon
A0A6J1WKV6	Aldehyde dehydrogenase, mitochondrial-like
A0A6J1WVD2	Carboxylesterase 1E
A0A6J1WUL1	Carboxylesterase 4A isoform X3
A0A6J1X0T9	Catalase
A0A6J1WBZ7	Dipeptidyl peptidase 3
A0A6J1WT08	FK506-binding protein 5 isoform X4
A0A6J1WEJ7	Glutathione peroxidase
A0A6J1W6I8	Glutathione S-transferase 1-1-like
A0A6J1X241	Glutathione S-transferase 1-1-like
A0A6J3BY34	Glutathione S-transferase 2-like
A0A6J1W9D2	Glutathione S-transferase-like
A0A6J1WGB7	glutathione transferase
A0A6J1X7Y3	HDHPR
A0A6J1X5C5	Heat shock 70 kDa protein cognate 4
A0A6J3C3E7	Heat shock 70 kDa protein cognate 5
A0A6J1WM77	Heat shock protein 60A-like
A0A3G1T1A3	Heat shock protein beta-1 isoform X2
A0A6J1WJM5	hydroxyacylglutathione hydrolase
A0A6J3C3B7	Hypoxia up-regulated protein 1
A0A6J1X8E0	Insecticyanin-A-like
A0A6J1WMF2	Lactoylglutathione lyase
A0A6J1WYU5	Leukocyte elastase inhibitor-like
A0A6J3C549	NAD(P)H oxidase (H2O2-forming)
A0A6J1WUV5	Peroxidase-like isoform X3
A0A6J3BT04	Peroxidase-like
A0A6J1WLZ7	Phosphotriesterase-related protein
A0A6J1WSZ9	Protein disulfide-isomerase
A0A6J3BWM0	Protein/nucleic acid deglycase DJ-1-like isoform X1
A0A6J1WTU4	Sphingomyelin phosphodiesterase
A0A6J1WGD3	Stress-induced-phosphoprotein 1-like
A0A6J3C787	Superoxide dismutase [Cu-Zn]
A0A6J1WJK9	superoxide dismutase
A0A6J3BWC0	Superoxide dismutase
A0A6J3C6F1	Thioredoxin reductase 1, mitochondrial
A0A6J3CAI3	thioredoxin-dependent peroxiredoxin
A0A6J1WKT1	Bleomycin hydrolase
A0A6J1WG05	carbonyl reductase (NADPH)
A0A6J1X8T4	Hydroxyacid oxidase 1
A0A6J3C9T9	Methanethiol oxidase
A0A6J1W702	Ommochrome-binding protein-like
A0A6J1WT18	Ommochrome-binding protein-like
A0A6J1X781	S-(hydroxymethyl)glutathione dehydrogenase
A0A6J3C2B6	Spermine oxidase-like
A0A6J1WGG6	Prostaglandin reductase 1
A0A3G1T1C0	Thioredoxin

Table S3.2b *G. mellonella* immune proteins detected in 96 hour infected larvae

**Aggregation**	
A0A6J3C5W3	Neuroglian-like
**AMP**	
A0A6J1WD07	27 kDa hemolymph protein
P85216	Anionic antimicrobial peptide 2
P85210	Cecropin-D-like peptide
A0A6J3BUX9	Defense protein Hdd11
A0A6J1WQI3	Gloverin-like
C9WHZ7	Lebocin-4
A0A6J1WBF9	Phosphatidylethanolamine-binding protein 1-like
A0A3G1T150	Seroin 3
A0A6J1X0P3	Seroin isoform X2
**Coagulation**	
A0A6J3C721	Hemocytin-like
A0A6J1WUY6	Scolexin B-like
**Fungal response**	
Q24995	Arylphorin
A0A6J1WVA9	Arylphorin subunit alpha-like
Q0E666	Beta-1,3-glucan-binding protein 1
A0A6J1WC08	Beta-1,3-glucan-binding protein-like isoform X2
A0A6J1WMZ6	Arylphorin subunit alpha-like
**Immune response**	
A0A6J1WV28	Cadherin-99C
A0A6J1WGG7	Transgelin
A0A6J1X611	Transgelin
A0A3G1T1R1	Calreticulin
A0A6J3CB97	5-hydroxytryptamine receptor 2C-like isoform X4
A0A6J3C4B5	Ninjurin-1 isoform X3
A0A6J1X8N5	Serine protease inhibitor 88Ea-like
A0A6J1W8W0	Thymosin beta isoform X1
A0A3G1T1A8	Thymosin
**Inflammation**	
A0A6J1W7W7	Cathepsin L
A0A6J1X834	Hemocyte protein-glutamine gamma-glutamyltransferase-like
A0A6J1X2Z4	Inter-alpha-trypsin inhibitor heavy chain H4-like isoform X6
A0A6J1X1F5	Leukotriene A-4 hydrolase isoform X2
A0A6J1X1Z8	Leukotriene A-4 hydrolase-like
A0A6J1WMA8	arginine kinase
**Iron Binding**	
A0A6J3C158	Transferrin-like isoform X1
A0A6J3BY59	Transferrin-like
A0A6J1W7V7	Transferrin
**Melanisation**	

A0A6J1W790	Serine protease inhibitor 77Ba-like
A0A6J1WSG2	tyrosinase
A0A6J1W8N1	Phenoloxidase-activating factor 2 (Fragment)
Q964D5	tyrosinase
**Phagocytosis**	
A0A6J1X347	Plastin-2
**Nutrient reservoir**	
A0A6J1WNC9	Apolipophorin-3
P80703	Apolipophorin-3
A0A6J1X726	Apolipophorins isoform X1
A0A6J1X7A0	Apolipophorins isoform X2
A0A6J1W9L8	Apolipoprotein D-like
**Pathogen binding**	
A0A6J1X8K1	Mucin-5AC
A0A6J1WNH2	Mucin-5AC-like
C7ASJ3	Hemolin
A0A6J3BX75	Spondin-1
A0A6J3C047	LOW QUALITY PROTEIN: apolipophorins-like
A0A6J1X3D5	N-acetylmuramoyl-L-alanine amidase
A0A6J1WTH3	Peptidoglycan recognition protein
